# Alkyl gallates inhibit serine *O*-acetyltransferase in bacteria and enhance susceptibility of drug-resistant Gram-negative bacteria to antibiotics

**DOI:** 10.3389/fmicb.2023.1276447

**Published:** 2023-10-27

**Authors:** Touya Toyomoto, Katsuhiko Ono, Tomoo Shiba, Kenta Momitani, Tianli Zhang, Hiroyasu Tsutsuki, Takeshi Ishikawa, Kanae Hoso, Koma Hamada, Azizur Rahman, Liping Wen, Yosuke Maeda, Keiichi Yamamoto, Masao Matsuoka, Kenjiro Hanaoka, Takuro Niidome, Takaaki Akaike, Tomohiro Sawa

**Affiliations:** ^1^Department of Microbiology, Graduate School of Medical Sciences, Kumamoto University, Kumamoto, Japan; ^2^Department of Applied Biology, Graduate School of Science and Technology, Kyoto Institute of Technology, Kyoto, Japan; ^3^Department of Chemistry, Biotechnology, and Chemical Engineering, Graduate School of Science and Engineering, Kagoshima University, Kagoshima, Japan; ^4^Faculty of Advanced Science and Technology, Kumamoto University, Kumamoto, Japan; ^5^Department of Hematology, Rheumatology, and Infectious Diseases, Faculty of Life Sciences, Kumamoto University, Kumamoto, Japan; ^6^Graduate School of Pharmaceutical Sciences, Keio University, Tokyo, Japan; ^7^Department of Environmental Medicine and Molecular Toxicology, Tohoku University Graduate School of Medicine, Sendai, Japan

**Keywords:** serine *O*-acetyltransferase, cysteine, persulfides, antimicrobial resistance, alkyl gallates, CysE inhibitors

## Abstract

A principal concept in developing antibacterial agents with selective toxicity is blocking metabolic pathways that are critical for bacterial growth but that mammalian cells lack. Serine *O*-acetyltransferase (CysE) is an enzyme in many bacteria that catalyzes the first step in l-cysteine biosynthesis by transferring an acetyl group from acetyl coenzyme A (acetyl-CoA) to l-serine to form *O*-acetylserine. Because mammalian cells lack this l-cysteine biosynthesis pathway, developing an inhibitor of CysE has been thought to be a way to establish a new class of antibacterial agents. Here, we demonstrated that alkyl gallates such as octyl gallate (OGA) could act as potent CysE inhibitors *in vitro* and in bacteria. Mass spectrometry analyses indicated that OGA treatment markedly reduced intrabacterial levels of l-cysteine and its metabolites including glutathione and glutathione persulfide in *Escherichia coli* to a level similar to that found in *E. coli* lacking the *cysE* gene. Consistent with the reduction of those antioxidant molecules in bacteria, *E. coli* became vulnerable to hydrogen peroxide-mediated bacterial killing in the presence of OGA. More important, OGA treatment intensified susceptibilities of metallo-β-lactamase-expressing Gram-negative bacteria (*E. coli* and *Klebsiella pneumoniae*) to carbapenem. Structural analyses showed that alkyl gallate bound to the binding site for acetyl-CoA that limits access of acetyl-CoA to the active site. Our data thus suggest that CysE inhibitors may be used to treat infectious diseases caused by drug-resistant Gram-negative bacteria not only via direct antibacterial activity but also by enhancing therapeutic potentials of existing antibiotics.

## 1. Introduction

Antimicrobial resistance (AMR) poses a major threat to human health around the world (Antimicrobial Resistance Collaborators, 2022). Recent study reported that 1.27 million deaths globally in 2019 were directly attributed to resistant bacteria and a further 4.9 million deaths were associated with bacterial AMR (Antimicrobial Resistance Collaborators, 2022). If no action is taken, the Review on Antimicrobial Resistance, commissioned by the UK government, argued that this figure could reach 10 million deaths per year by 2050, thus surpassing cancer, diabetes, and heart disease as the leading cause of death worldwide (O’Neill, 2014). It is thus clear that the need for new antibacterial drugs to treat the increasing global prevalence of drug-resistant bacterial infections. According to the analysis by Butler et al., 62 new antibiotics are currently under development, including 47 direct-acting antibacterials, 5 non-traditional small molecule antibacterials, and 10 β-lactam/β-lactamase inhibitor combinations (Butler et al., 2023). Compounds with novel mechanisms of action or molecular targets, or those belonging to new classes of drugs are particularly needed.

Blocking metabolic pathways that are critical for bacterial growth but that mammalian cells are missing is a key approach to developing antibacterial agents with selective toxicity. The sulfur-containing amino acid l-cysteine is essential for growth of many organisms because it is a major constituent of proteins, antioxidants, cofactors, vitamins, and metal clusters (Leustek et al., 2000; Kessler, 2006). Many bacteria biosynthesize l-cysteine from l-serine via two-step enzymatic reactions (Supplementary Figure S1; Verma and Gupta, 2021). In the first step, l-serine is converted to *O*-acetylserine (OAS), with acetyl coenzyme A (acetyl-CoA) used as a co-substrate, by means of the catalytic action of serine *O*-acetyltransferase (CysE). In the second step, OAS is converted to l-cysteine, the reaction being catalyzed by *O*-acetylserine sulfhydrylase (CysK). In mammalian cells, l-cysteine is biosynthesized from l-homocysteine via intermediate formation of cystathionine, as catalyzed by cystathionine β-synthase and cystathionine γ-lyase (Verma and Gupta, 2021). Lack of the CysE/CysK-dependent l-cysteine biosynthesis pathway in mammals suggests that those bacterial enzymes may become targets for antimicrobial agents (Verma et al., 2020; Verma and Gupta, 2021).

CysE in bacterial viability and pathogenesis is important for several bacteria including *Escherichia coli* (Zhang and Lin, 2009), *Haemophilus influenzae* (Akerley et al., 2002), *Staphylococcus aureus* (Chaudhuri et al., 2009), and *Bacillus subtilis* (Kobayashi et al., 2003). CysE also reportedly plays an essential role in the survival of *Mycobacterium tuberculosis* during the persistent phase of infection *in vivo* (Sassetti et al., 2003; Sassetti and Rubin, 2003; Rengarajan et al., 2005). *Serratia marcescens*, an opportunistic pathogen, requires CysE for flagellar transport and phospholipase/lecithinase activity (Anderson et al., 2017). These findings have motivated researchers to develop CysE inhibitors as a new class of antimicrobial agents (Verma et al., 2020; Verma and Gupta, 2021). In this context, Chen et al., reported that several natural compounds that could inhibit the enzyme activity of recombinant CysE suppressed the growth of *S. aureus* and had antibiofilm effects (Chen et al., 2019). Magalhaes et al., similarly reported that synthetic compounds that possessed CysE inhibitory activities had antibacterial effects against *Salmonella* (Magalhães et al., 2020). In those studies, CysE inhibition was confirmed by means of *in vitro* assays that used recombinant enzymes. However, whether those compounds could inhibit CysE inside bacteria and reduce intrabacterial levels of l-cysteine and related metabolites such as glutathione (GSH) was not determined. In our study here, we aimed at finding CysE inhibitors from a chemical library (100,000 compounds) by developing a high-throughput screening system. We determined that alkyl gallates, particularly octyl gallate (OGA), showed potent CysE inhibitory activities. OGA has long been used as a food additive to prevent oxidation of lipid (Qiu et al., 2022). It has also been reported the antibacterial activity of OGA against Gram-positive bacteria including methicillin resistance *S. aureus* (MRSA) and *Bacillus subtilis*. In addition to those effects of OGA, this study clearly demonstrated that OGA could sensitize bacteria against oxidative stress by reducing antioxidant molecules such as GSH and its persulfide (glutathione persulfide; GSSH). More important point is that OGA could markedly enhance the antibiotic susceptibilities of clinical isolates of multidrug-resistant *E. coli* and *Klebsiella pneumoniae*. Our findings indicate that blockage of CysE activity may become potential strategy to treat infections from drug-resistant Gram-negative bacteria, both through their specific antibacterial activity and amplifying the efficacy of current antibiotics.

## 2. Materials and methods

### 2.1. Bacterial strains

*E. coli* BW25113 (wild type) and single-gene disruption mutants (*cysE, fliY, ydjN*, and *katG*) were obtained from the National Institute of Genetics, Shizuoka, Japan. To construct the *fliY*/*ydjN* double mutant, P1 phage-mediated transduction was used to introduce the mutation *fliY::Kan* into the *ydjN* mutant by following a general protocol (Thomason et al., 2007).

### 2.2. Clinical isolates of bacteria

Two clinically isolated *E. coli* strains (C.I *E. coli*-1 and C.I *E. coli*-2) and one C.I *K. pneumoniae* strain were used. C.I *E. coli-1* was used for flow cytometry assay as described in the section 3.14. C.I *E. coli*-2 and C.I *K. pneumoniae* are multi-drug resistance (as shown in Supplementary Tables S1, S2) that were used for antibiotics susceptibility test in the presence or absence of OGA, as described in section 3.15.

### 2.3. PCR determination of IMP-1

After confirming that both C.I *E. coli*-2 and C.I *K. pneumoniae* used were positive for the modified carbapenem inactivation method, we searched for resistance genes by using the Cika Geneus® Carbapenemase Genotype Detection Kit 2 (Kanto Chemical Company). For carbapenemase-producing genes, we used the *bla*_IMP-1 group_, *bla*_VIM group_, *bla*_GES group_, *bla*_KPC group_, *bla*_NDM group_, *bla*_OXA-48 group_, and *bla*_IMP-6_ according to the reagent’s package insert with Multiplex PCR. As result, these strains expressed only the *bla*_*IMP-1* group_.

### 2.4. Bacterial culture

All bacteria used in this study were subcultured in LB medium [1% NaCl (Nacalai Tesque), 1% peptone (Nihon Seiyaku), 0.5% yeast extract (Oriental Yeast Company, Ltd.)]. M9 minimal medium [33 mM Na_2_HPO_4_ (FujiFilm Wako Pure Chemical Corporation, Ltd.), 22 mM KH_2_PO_4_ (Nacalai Tesque), 8.6 mM NaCl, 9.4 mM NH_4_Cl (FujiFilm Wako Pure Chemical Corporation, Ltd.), 0.1 mM CaCl_2_ (FujiFilm Wako Pure Chemical Corporation, Ltd.), and 1 mM MgSO_4_ (Nacalai Tesque)] supplemented with 0.2% glucose (Glc) (FujiFilm Wako Pure Chemical Corporation, Ltd.) with or without 0.2% casamino acid (CA) (Becton, Dickinson and Company) were using for inhibitor screening (M9 + Glc or M9 + Glc + CA). Detail conditions are described later in section 3.7. All other experiments including flow cytometry (section 3.14) and antibiotic sensitivity (section 3.15) were performed by using M9 + Glc + *CA.*

### 2.5. Materials

Serine, NaHS, acetyl-CoA trilithium salt, ampicillin, imidazole, and dimethyl sulfoxide (DMSO) were purchased from FujiFilm Wako Pure Chemical Corporation, Ltd. Monobromobimane and OGA was from Tokyo Chemical Industry Company, Ltd. Isopropyl-β-d-thiogalactopyranoside and lysozyme were from Nacalai Tesque. β-(4-Hydroxyphenyl)ethyl iodoacetamide (HPE-IAM) was purchased from Molecular Biosciences (Boulder). Tween 20 was purchased from Sigma Aldrich. Formic acid (FujiFilm Wako Pure Chemical Corporation, Ltd.) and acetonitrile (Kanto Chemical Company) were used as the mobile phase for liquid chromatography-mass spectrometry (LC–MS) and LC–MS/MS analyses. Gallic acid was purchased from Sigma Aldrich. Gallic acid derivatives (2-hydroxybenzoic acid, 3-hydroxybenzoic acid, 4-hydroxybenzoic acid, 2,3-hydroxybenzoic acid, 3,4-hydroxybenzoic acid, and 3,5-hydroxybenzoic acid) and alkyl gallates (ethyl gallate, butyl gallate (BGA), isoamyl gallate, OGA, dodecyl gallate, hexadecyl gallate, and stearyl gallate) were from Tokyo Chemical Industry Company, Ltd.

### 2.6. Purification of CysE recombinant enzyme

*N*-Terminally His-tagged CysE from *Salmonella enterica* serovar Typhimurium LT2 was expressed and purified in the *E. coli* system as described previously (Ono et al., 2017). In brief, *E. coli* BL21(DE3) pLysS harboring pQE80L-CysE was cultured in LB medium supplemented with 100 μg/mL ampicillin at 30°C until optical density at 660 nm reached 0.5. Isopropyl-β-d-thiogalactopyranoside (0.5 mM) was added to the culture and inoculated at 30°C for 1 h. After we centrifuged the culture, we washed the bacterial pellet with saline and then suspended it in lysis buffer (50 mM sodium phosphate buffer pH 8.0, 300 mM NaCl, and 10 mM imidazole) containing 1 mg/mL lysozyme and 0.1 μg/mL DNase I (Qiagen). The bacterial suspension was subjected to sonication and centrifuged. Supernatants were applied to an Ni-NTA agarose column (Qiagen), and the column was washed twice with washing buffer (50 mM NaPB pH 7.4, 300 mM NaCl, and 20 mM imidazole). His-tagged CysE was eluted with 250 mM imidazole in lysis buffer, and the sample was then subjected to a gel filtration column (PD MiniTrap G-25; GE Healthcare) to replace the solvent with storage buffer (50 mM NaPB pH 7.4). Purified StCysE was analyzed by sodium dodecyl sulfate-polyacrylamide gel electrophoresis (Supplementary Figure S2).

### 2.7. High-throughput screening of CysE inhibitors

A screening chemical library containing 102,400 compounds was provided by the Drug Discovery Initiative (DDI), at The University of Tokyo. For first screening, we used a CysE enzyme inhibition assay with a 384-well plate format, as performed with following protocols: 10 μL of CysE solution (0.02 μg/mL CysE, 2 mM serine, 0.005% Tween-20, and 100 mM NaPB, pH 7.4) was added to the 384-well plates (black, non-binding, flat-bottom, #784900, Greiner Bio-One) containing 0.25 μL of the chemical library (25 μM final concentration) by means of Multidrop Combi (Thermo Fisher Scientific), after which the plates were incubated at room temperature for 1 h, and then 10 μL of acetyl-CoA (200 μM) was added to the 384-well plates. Incubation continued at room temperature for 1 h, and then 1 μL of MBB (600 μM) was added to the plates and incubation continued at room temperature for 1 h. The CysE reaction mixture reacted with DMSO and the CysE solution without CysE enzyme were used as positive and negative controls, respectively. The fluorescence intensity of the CoA-bimane adduct, the reaction product of CysE, was detected by using a fluorescence microplate reader (excitation at 430 nm/emission at 530 nm; PHERAstar Plus, BMG Labtech) according to the manufacturer’s instructions. The CysE inhibition ratio of the chemical library was calculated by means of a Microsoft Excel macro-based program provided by the DDI. The compounds that showed a CysE inhibition of >70% (1,040 compounds) were studied for dose dependence (1.5, 3.125, 6.25, 12.5, and 25 μM) and reproducibility by using the same protocol as that used in the first screening. The 500 compounds that showed strong inhibition of CysE were subjected to an antibacterial assay (first antibacterial assay). An overnight cultures of *E. coli* BW25113 in LB medium was diluted 1,000-fold by using M9 + Glc or M9 + Glc medium supplemented with 100 μM cystine (M9 + Glc + cystine). The *E. coli* cultures (50 μL) were transferred to 96-well plates (non-coating, clear, #MS-8096R, Sumitomo Bakelite Company, Ltd.) containing 1 μL of the chemical library (final concentration of 40 μM), and they were then incubated at 37°C for 24 h. The optical density at 660 nm was measured by using a microplate reader (iMark, Bio-Rad). Bacterial cultures containing DMSO or containing only medium (without bacteria) were used as positive and negative controls, respectively. The 43 compounds that did not show antibacterial activity in M9 + Glc + cystine but did show it in M9 + Glc were chosen as candidates for cysteine synthase-specific inhibitors and subjected to the second antibacterial assay. This second antibacterial assay was performed by using three *E. coli* strains, wild type, *cysE,* and *fliY*/*ydjN* mutants (cystine transporter deletion mutants) via the same method as the first antibacterial assay except that M9 + Glc + CA was used as the medium. The antimicrobial activity of the 43 compounds was tested against three *E. coli* strains, and two compounds (compounds A and B) showed strong antimicrobial activity against only the *fliY*/*ydjN* mutant but not the wild type and the *cysE* mutant. Dose dependency of antimicrobial activity of two compounds were performed by using wt and *fliY*/*ydjN* mutant.

### 2.8. Antibacterial assays of gallic acid derivatives

We determined the antibacterial activities of gallic acid derivatives by means of the serial dilution method. The overnight cultures of *E. coli* BW25113 in LB medium were diluted 1,000-fold in M9 + Glc + *CA.* The suspensions of *E. coli* culture (100 μL) were plated in a 96-well plate (0.1 mL/well) and were treated with various concentrations of gallic acid derivatives. The DMSO concentration was adjusted to 1% in all assays. After incubation at 37°C overnight, we measured the optical density at 655 nm with a microplate reader to determine bacterial growth.

### 2.9. LC–MS analyses

Compounds used in this study were analyzed by means of LC–MS. We used LC-electrospray ionization (ESI)-MS with the Agilent 6,460 Triple Quadrupole LC/MS System (Agilent Technologies). Samples were injected into a YMC-Triart C18 Plus column (2.1 × 50 mm) (YMC Co., Ltd.) at 45°C and were then separated by using gradient programs with mobile phases A (H_2_O + 0.1% formic acid) and B (acetonitrile) (B concentration), 0 min—5%, 10 min—80%, 10.1 min—5%, and 15 min—5%. General conditions used for ESI-MS were as follows: nebulizer gas, nitrogen delivered at 50 psi; nebulizer gas temperature, 250°C; capillary voltage, 3,500 V; and collision gas, G1 grade nitrogen (Taiyo Nippon Sanso Corporation).

### 2.10. LC–MS/MS quantification of intracellular thiols in bacteria treated with OGA

Overnight cultures of *E. coli* BW25113 were diluted 1,000-fold in M9 + Glc + CA medium with added 30 μM OGA, followed by incubation at 37°C for 4 h with shaking. Bacterial cultures were centrifuged to obtain bacterial pellets, and pellets were washed once with saline. OGA-treated bacterial pellets were suspended in methanol containing 5 mM HPE-IAM (Akaike et al., 2017). The suspensions were subjected to sonication (high output for 2 min) via the Bioruptor (Cosmo Bio Company, Ltd.), and then they were incubated at 37°C for 15 min. Insoluble precipitates were removed by centrifugation, and supernatants were diluted 10 times by using 0.1% formic acid. HPE-derivatized thiol molecules were quantitated by means of LC–MS/MS as reported previously (Zhang et al., 2019; Ono et al., 2021).

### 2.11. LC–MS/MS quantification of gallic acid and OGA

Overnight cultures of *E. coli* BW25113 were diluted to an optical density at 600 nm = 1.0 by using M9 + Glc + *CA.* To this culture, we added 100 μM gallic acid or OGA followed by incubation at 37°C for 15 min. *E. coli* cells were washed by using phosphate-buffered saline (PBS) twice and were suspended in methanol. After sonication by using the Bioruptor, cells were centrifuged to remove the insoluble fraction, and collected supernatants were diluted 5 times with 0.1% formic acid. Gallic acid and OGA were analyzed by means of LC–MS/MS with use of the MRM parameters as follows: gallic acid: precursor ion (*m/z*), 169.0; product ion (*m/z*), 125.0; fragmentor voltage (V), 90; collision energy (V), 13; polarity, negative. OGA: precursor ion (*m/z*), 281.1; product ion (*m/z*), 124.0; fragmentor voltage (V), 130; collision energy (V), 29; polarity, negative.

### 2.12. Preparation of amino acid standards

Different amino acids (5 mM) were reacted with 5 mM 3-aminopyridyl-*N*-hydroxysuccinimidyl carbamate (APDS) (APDSTAG, 014–23,841; FUJIFILM Wako Pure Chemical Corporation, Ltd.) in 100 mM boric acid (pH 8.9) at 60°C for 30 min, according to the literature (Shimbo et al., 2009). The reaction mixtures were subjected to preparative high-performance liquid chromatography (HPLC) for purification of APDS-amino acid adducts. Preparative HPLC was performed by using the Agilent 1,260 Infinity series equipped with a photodiode array detector and an automated fraction collector (Agilent Technologies). Samples including APDS-aspartic acid and APDS-histidine were separated on a YMC-Triart C18 column (250 × 4.6 mm inner diameter) (YMC Company Ltd.), and other APDS-amino acid adducts were separated on a YMC-Pack ODS-AQ column (250 × 4.6 mm inner diameter) (YMC Company Ltd.) at 35°*C. mobile* phases A (H_2_O + 0.1% formic acid) and B (acetonitrile) were used with a linear gradient of from 0.5 to 30% B in 16 min, with the gradient maintained at 30% B for 1 min, after which the gradient was reduced to 0.5% B for 1 min, with a flow rate of 0.2 mL/min. Those APDS-amino acids were detected at 210 nm. Formation of APDS-amino acids was confirmed by means of mass spectrometry as described below. Peaks corresponding to APDS-amino acids were collected by using a fraction collector and were subjected to lyophilization.

### 2.13. Quantification of bacterial amino acids

On the basis of the reactions between APDS and amino acids, we used the LC-ESI-MS with the Agilent 6,460 Triple Quadrupole LC–MS system (Agilent Technologies) to determine the amino acid levels in bacterial cells (Shimbo et al., 2009). In brief, treated bacteria were washed twice with PBS and resuspended in 90 μL of 100 mM boric acid (pH 8.9). Those samples were homogenized by using the Bioruptor UCD-250 for 10 min, followed by collecting supernatants via centrifugation at 13,000 rpm and 4°C for 10 min. The supernatants were then mixed with 10 μL of 100 mM APDS. After incubation at 60°C for 30 min, some portions of the mixtures were diluted 5 times with 100 mM boric acid (pH 8.9), after which the samples were used to determine the protein concentrations with the BCA Protein Assay Kit (FUJIFILM Wako Pure Chemical Corporation, Ltd.). Simultaneously, other portions of the mixtures were diluted 10 times with methanol, and the precipitates were removed by centrifugation under the same conditions. The supernatants were again diluted 10 times with water, after which they were subjected to LC–MS/MS analysis. LC–MS/MS conditions were as follows: column, Wakopak Ultra APDS TAG® column (2.1 × 100 mm inner diameter) (FUJIFILM Wako Pure Chemical Corporation, Ltd.); column temperature, 40°C; injection volume, 3 μL; mobile phases: A, 0.1% formic acid (pH 6.0, adjust with ammonium hydrogen carbonate), and B, 60% acetonitrile in H_2_O; gradient (B concentration), 0 min—1%, 1 min—1%, 10 min—30%, 10.1 min—1%, 25 min—1%; and flow rate, 0.2 mL/min. The general conditions for ESI-MS were nebulizer gas, nitrogen, delivered at 50 psi; nebulizer gas temperature, 250°C; capillary voltage, 3,500 V; collision gas, and G1 grade, nitrogen (Taiyo Nippon Sanso Corporation). Supplementary Table S3 provides details of the MRM parameters that we used in this study.

### 2.14. Flow cytometry determination of H_2_O_2_ in bacteria

Wild type *E. coli* BW25113, C.I *E. coli*-1 and *katG* disruption mutant were cultured overnight in LB medium with shaking. Cells were diluted 1,000-fold in M9 + Glc + CA medium and treated with 30 μM OGA for 4 h under static conditions or were untreated. After dilution (optical density at 660 nm was adjusted to approximately 0.1), cells were treated with 10 μM HYDROP (Goryo Chemical) for 90 min in dark. After the cells were washed twice with PBS, they were treated with 1 mM H_2_O_2_ for 30 min or were untreated. The fluorescence intensity of the bacteria was determined by means of the BD FACSCalibur flow cytometer (BD Biosciences). Dot plot data were analyzed by using the FlowJo FACS analysis software ver. 10.8.1 (BD Biosciences).

### 2.15. Alterations of antibiotics sensitivity in the presence of OGA

C.I *E. coli*-2 and C.I *K. pneumoniae* were cultured overnight in LB medium with shaking. Cells were diluted 1,000-fold in M9 + Glc + CA medium then treated with several types of antibiotics in the presence or absence of 30 μM OGA in shake condition. After 24 h incubation, bacterial growth was measured by 655 nm absorbance.

### 2.16. Antibiotic susceptibility test

We used the antibiotic susceptibility test for C.I *E. coli*-2 and C.I *K. pneumoniae* by means of ready-state Dry Plates (DP-45) (Eiken Chemical Company). The plate contained 22 antimicrobial agents, including piperacillin, tazobactam/piperacillin, cefepime, ceftazidime, ceftazidime/DPA, cefozopran, gentamycin, minocycline, doripenem, amikacin, levofloxacin, aztreonam, imipenem, imipenem/DPA, meropenem, meropenem/DPA, colistin, tobramycin, ciprofloxacin, sulfamethoxazole/trimethoprim, sulbactam/cefoperazone, and fosfomycin. The overnight cultures of *E. coli* and *K. pneumoniae* were diluted 1:1,000 in Mueller Hinton Broth (Becton Dickinson and Company). A 100-μL sample of the suspension was added to each well and incubated for 20 h at 37°C. The MIC values of each antibiotic were determined by visual observation. Susceptibility to the antibiotics was classified as sensitive (S), intermediate (I), or resistant (R) according to the MIC values (CLSI M100-S22 standard) defined by the Clinical and Laboratory Institute (CLSI).

### 2.17. Crystallization of StCysE

Crystallization conditions were screened by using the sitting-drop vapor-diffusion method with the reservoir solutions supplied in commercially available screening kits (Crystal Screen, Crystal Screen 2, and PEG-ION 1/2, and PEGRx 1/2, SaltRx 1/2, and Grid Screen PEG6000 from Hampton Research, and Wizard Classic 1, 2, 3, and 4 and Wizard Cryo 1 and 2 from Emerald BioSystems). A droplet made by mixing 1.0 μL of purified StCysE (5 or 10 mg/mL) with an equal volume of a reservoir solution was equilibrated against 100 μL of the reservoir solution at 293 K. Crystals of the cysteine complex with CysE appeared 2 days after the reservoir conditions: 5 mg/mL StCysE, 5 mM cysteine, 5 mM dithiothreitol (DTT), 100 mM NaCl, and 0.1 M HEPES-NaOH, pH 7.2. Crystals of the serine complex with CysE appeared 3 days after the reservoir conditions: 10 mg/mL StCysE, 10 mM serine, 5 mM DTT, 400 mM NaCl, and 0.1 M HEPES-NaOH, pH 7.2. Crystals of the CoA complex with CysE appeared 3 days after the reservoir conditions: 10 mg/mL StCysE, 100 mM CoA, 5 mM cysteine, 5 mM DTT, and 0.15 M potassium sodium tartrate. Crystals of the BGA complex with CysE appeared 3 days after the reservoir conditions: 10 mg/mL StCysE, 5 mM BGA, 5 mM cysteine, 5 mM DTT, 15%, 2-methyl-2,4-pentanediol, 100 mM NaCl, and 0.1 M HEPES-NaOH, pH 7.2. Before being flash-cooled in liquid nitrogen, the crystals were soaked in reservoir solution supplemented with 20% glycerol as a cryoprotectant.

### 2.18. Data collection and structure determination

All crystal data were collected at a wavelength of 0.90000 Å under gaseous nitrogen (100 K) on the synchrotron radiation beamline BL44XU at the SPring-8 facility (Harima, Japan). All diffraction data were processed and scaled with XDS and XSCALE (Kabsch, 2010). Supplementary Table S4 summarizes the statistics for data collection and processing. We determined the crystal structure of the cysteine complex with StCysE by the molecular replacement method and using the structure of EcCysE as a search model (PDB code: 1T3D). We determined all the crystal structures for the StCysE-compound complex by using the molecular replacement method with MORLEP (Vagin and Teplyakov, 1997), with the Cys complex form of StCysE as a search model. Manual model rebuilding and crystallographic refinement of StCysE complex structures were performed via Coot (Emsley and Cowtan, 2004) and REFMAC5 (Murshudov et al., 1997). Supplementary Table S4 summarizes the refinement statistics.

### 2.19. Inhibitory kinetics analysis

We determined the enzymatic kinetics of CysE by using various concentrations of acetyl-CoA, as reported previously (Chen et al., 2019). In this study, 25 or 50 μM of BGA was added to 10 μg/mL StCysE in 100 mM NaPB (pH 7.6) and the samples were incubated at 37°C for 1 min. l-Serine (5 mM) and AcCoA (0.1–5 mM) were then reacted with StCysE at 37°C for 1 min, after which 0.1% of formic acid was added to the sample to terminate the enzymatic reactions. The samples were subjected to LC–MS/MS to measure the production of OAS. The data were plotted by (nonlinear regression curve fitting) by using GraphPad Prism 6.0. Kinetic parameters were determined by means of a Lineweaver-Burk plot.

### 2.20. Statistical analysis

We used Student’s *t*-test to determine significant differences when only two treatment groups were being compared. All data are given as means ± standard deviation (SD). Data for each experiment were acquired from at least three experiments. A *p-*value of less than 0.05 was said to be statistically significant.

## 3. Results

### 3.1. High-throughput screening system

**Figure 1:**
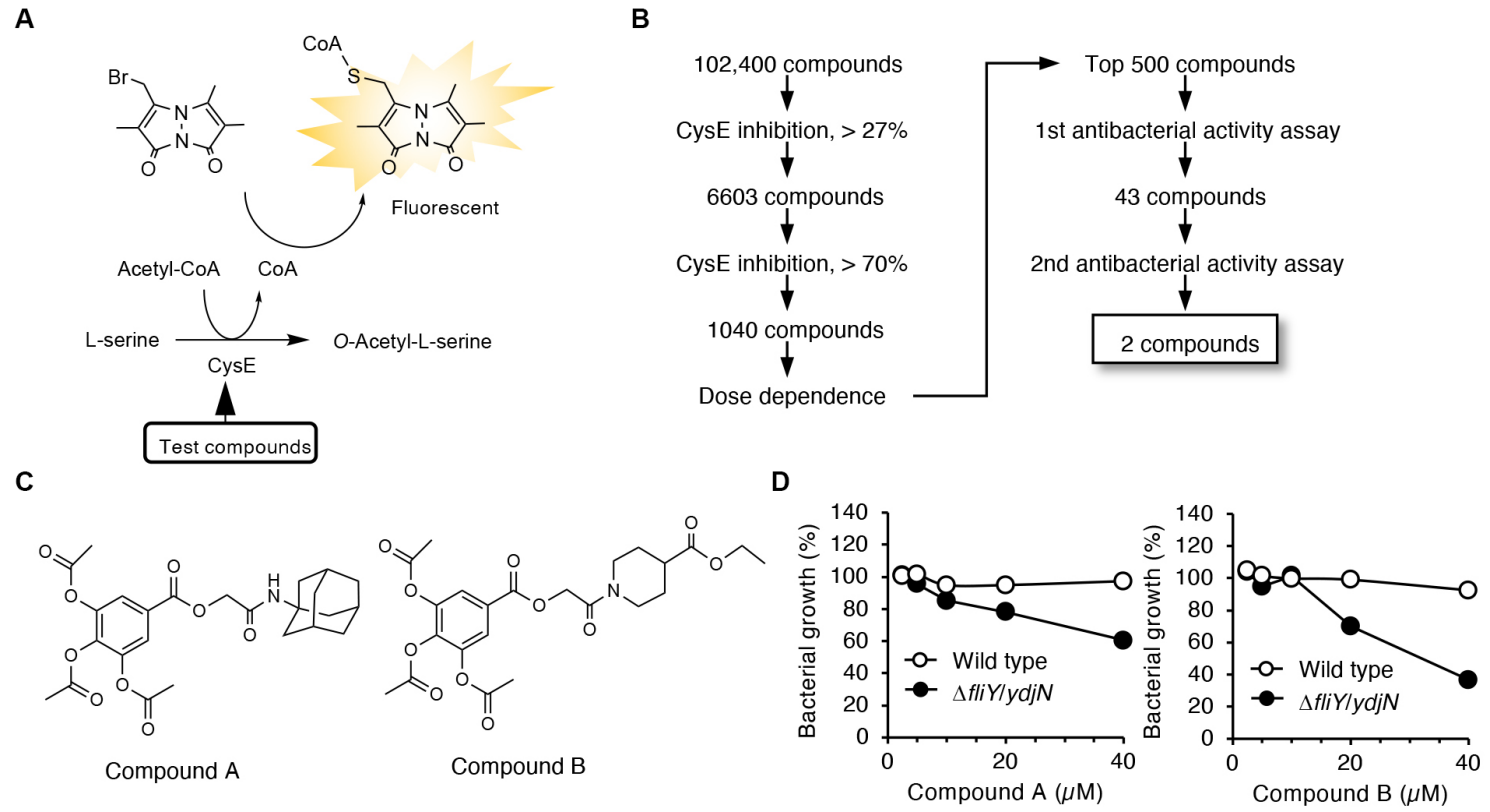
High-throughput screening system for identification of CysE inhibitors. **(A)** Design of the first screening process. CoA produced from the CysE reaction was detected by using the thiol-reactive reagent MBB. Formation of CoA-MBB conjugates was determined by means of fluorescence spectrometry. Compounds that reduced fluorescence intensities were screened. **(B)** Flowchart for the first and second screenings. **(C)** Chemical structures of the two compounds screened. **(D)** Antibacterial activities of these two compounds. Growth of the wild-type and the cystine transporter mutant in the presence of the indicated concentrations of compounds A and B.

We developed a high-throughput screening system to identify compounds that possess both CysE inhibitory activities and antibacterial activities. In the first screening, compounds that can inhibit enzymatic reactions catalyzed by recombinant CysE were screened by detecting CoA as a reaction product when using the thiol-reacting fluorophore MBB (Figure 1A). In this study, we used recombinant CysE cloned from *Salmonella Typhimurium* (StCysE) (Ono et al., 2017). We investigated 102,400 compounds for their effects on fluorescence intensities produced during the enzymatic reactions. As Figure 1B shows, the top 500 compounds were subjected to a second screening. In the second screening, the antibacterial activities of those compounds were determined by using two *E. coli* strains, wild-type *E. coli* and *E. coli* lacking cystine transporters, FliY/YdjN (a cystine transporter mutant). Growth of the cystine transporter mutant depends on endogenous cysteine biosynthesis (Ohtsu et al., 2015). Thus, growth inhibition by CysE inhibitors should be more obvious for the cystine transporter mutant than for the wild-type *E. coli*. On the basis of this expectation, we studied the antibacterial activities of 500 compounds and found that 2 compounds (compounds A and B; Figure 1C) showed a clear difference in terms of antibacterial activities against wild-type *E. coli* and the *E. coli* cystine transporter mutant FliY/YdjN (Figure 1D).

### 3.2. CysE inhibitory activities and antibacterial activities of gallic acid derivatives

**Figure 2:**
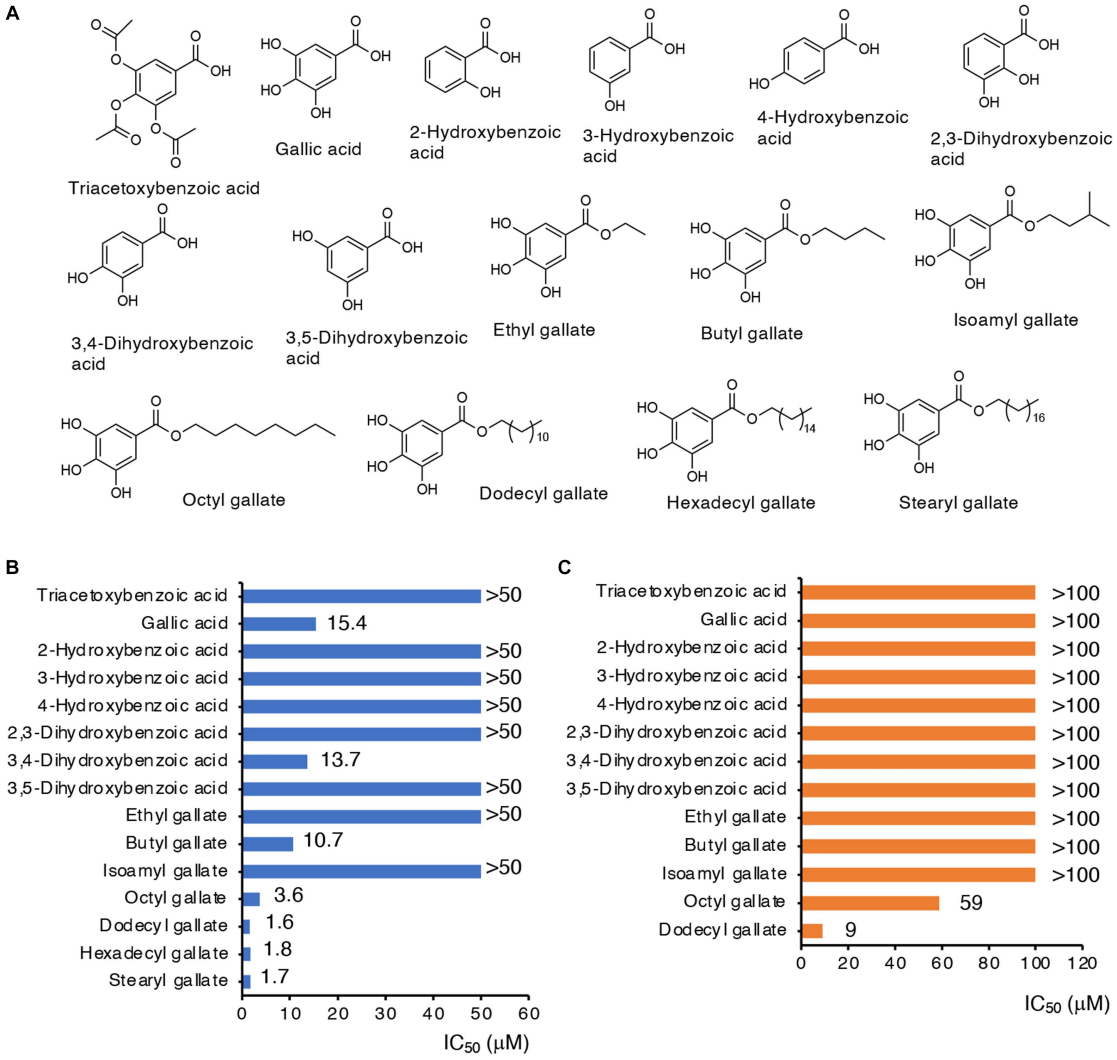
CysE inhibitory activities and antibacterial activities of gallate derivatives. **(A)** Chemical structures of gallate derivatives examined in this study. **(B)** CysE inhibitory activities of gallate derivatives. Effects of gallate derivatives on CysE reaction were determined. **(C)** Antibacterial activities of gallate derivatives.

Both compounds A and B contain a triacetoxybenzoate moiety in their structures (Figure 1C). We next studied whether this moiety was essential for CysE inhibition and antibacterial activity. Triacetoxybenzoic acid itself did not show CysE inhibition or antibacterial activity (Figure 2). We then investigated the CysE inhibitory activity of gallic acid, which can be formed by hydrolytic removal of acetoxy residues from triacetoxybenzoic acid. As seen in Figure 2B, we clearly observed CysE inhibitory activity by gallic acid. We continued our investigation of the possible requirement of hydroxyl moieties in benzoate for CysE inhibition by using mono- and dihydroxybenzoic acid derivatives. Among the hydroxybenzoic acid derivatives examined, only gallic acid and 3,4-dihydroxybenzoic acid showed CysE inhibition (Figure 2B). Both gallic acid and 3,4-dihydroxybenzoic acid failed to show antibacterial activities, however (Figure 2C). We then studied the effect of side chains on the gallic acid moiety. The introduction of alkyl chains to gallic acid enhanced CysE inhibitory activities, except for ethyl and isoamyl side chains (Figure 2B). Notably, alkyl gallates showed potent antibacterial activities (Figure 2C). These data suggest that both the gallic acid moiety and the hydrophobic side chain are essential for CysE inhibition and antibacterial activity. We hypothesized that the highly hydrophilic gallic acid could not enter the bacterial cytosol where the CysE reaction took place. To determine the bacterial uptake of gallic acid and its alkylated derivatives, we utilized LC–MS/MS with MRM (Supplementary Figure S3). As shown in Supplementary Figures 3E,F, only a trace amount of gallic acid was detected in bacterial extracts. In contrast, bacterial uptake of OGA was 4,360 times higher than that of gallic acid (Supplementary Figures 3E,F). Insufficient antibacterial activity observed for gallic acid may be ascribed, at least in part, to this low accessibility to cytosol. Gallic acid and its alkylated derivatives have been known for their antibacterial activities. In those studies, alkyl gallates showed more potent antibacterial activities than did gallic acid. Bacteria that were susceptible to gallate derivatives included *Salmonella Choleraesuis* (Kubo et al., 2002), *B. subtilis* (Kubo et al., 2004), *E. coli*, *Vibrio parahaemolyticus*, *Listeria monocytogenes* (Shi et al., 2021), and *S. aureus* (Shi et al., 2021). Whether alkyl gallates could inhibit CysE in bacteria and reduce intrabacterial levels of cysteine and its related metabolites remained unknown. Therefore, in the next experiments, we used OGA as a representative alkyl gallate and investigated the effects of OGA on intracellular cysteine production in *E. coli*.

### 3.3. Effects of OGA treatment on intrabacterial levels of cysteine, GSH, their persulfides, and other amino acids

**Figure 3:**
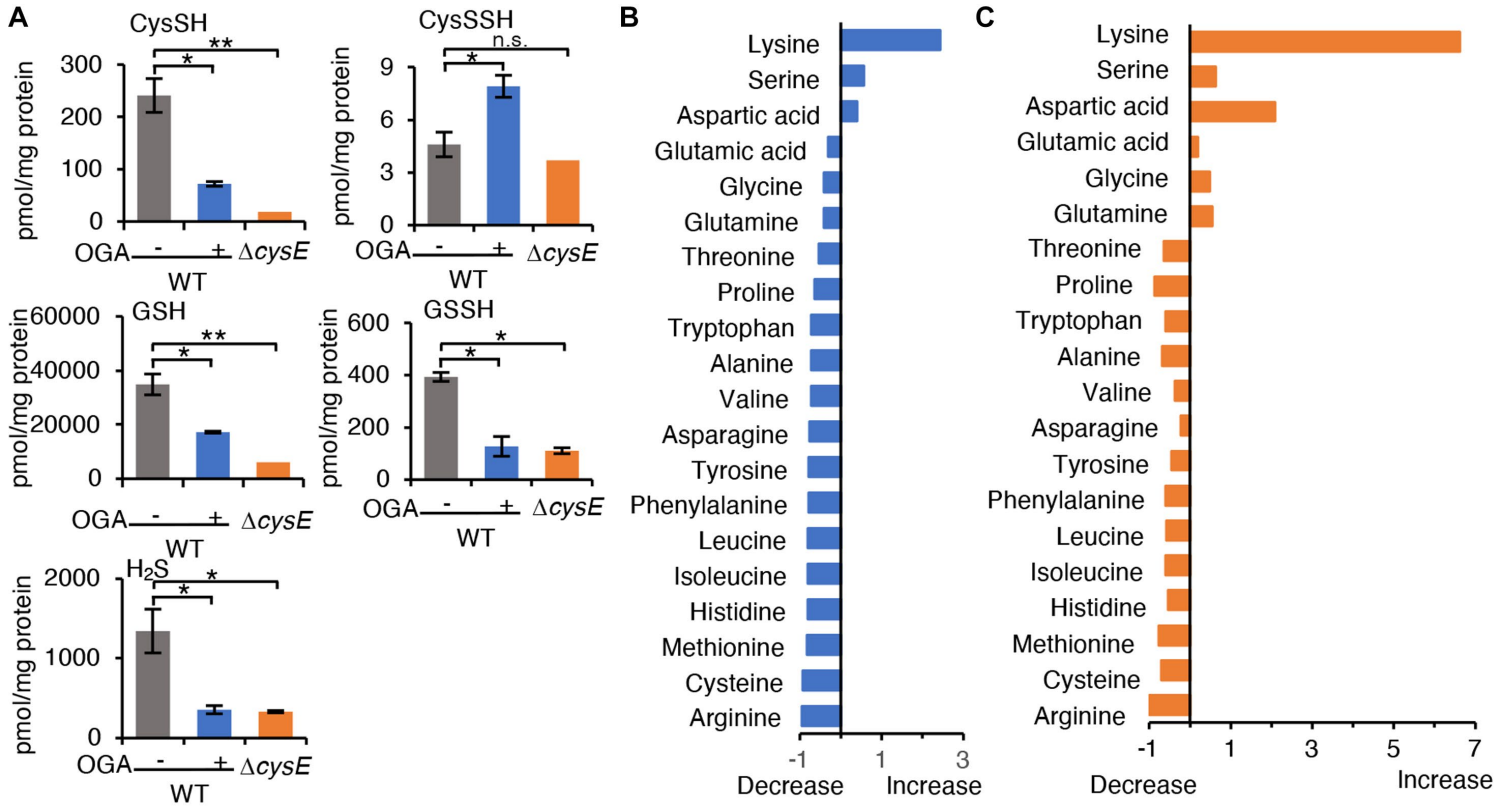
Metabolomic analysis. **(A)** Cysteine and its related molecules in *E. coli*. Wild-type *E. coli* treated with OGA (30 μM) or untreated and the cystine transporter mutant were analyzed via LC–MS/MS metabolomics with the use of thiol-alkylating agent HPE-IAM. Data are means ± SD (*n* = 3). *p < 0.05; **p < 0.01; n.s., not significant. **(B,C)** Amino acid levels in *E. coli* were determined by using LC–MS/MS after APDS derivatization. Alterations in amino acid concentrations were plotted as changes against wild-type *E. coli*. Supplementary Figure S6 shows individual amino acid concentrations.

To examine whether OGA treatment indeed resulted in inhibition of CysE in bacteria, we measured intrabacterial levels of cysteine, GSH, and their persulfides, after OGA treatment or no treatment, by means of LC–MS/MS with HPE-IAM derivatization method (Akaike et al., 2017). The concentration of OGA (30 μM) that induced approximately 30% growth inhibition was used. As Figure 3A illustrates, OGA treatment markedly reduced cysteine levels in wild-type *E. coli* to a degree that was comparable to that in the *cysE* mutant. In addition, we found a significant reduction in GSH in wild-type bacteria after OGA treatment. Cysteine and GSH reportedly exist in their persulfide forms, i.e., cysteine persulfide (CysSSH) and GSSH, in both prokaryotes and eukaryotes (Ida et al., 2014; Akaike et al., 2017; Sawa et al., 2020; Ono et al., 2021). Marked reduction in GSSH was also observed in OGA-treated *E. coli*, similar to that found in the *cysE* mutant. We also noted a reduction in hydrogen sulfide (H_2_S) in *E. coli* after OGA treatment. These data suggest that OGA may inhibit CysE in bacteria, which would result in reduced levels of intracellular cysteine and its related molecules including GSH, GSSH, and H_2_S. We also examined the effects of quercetin on intrabacterial levels of cysteine and related metabolites because of its known CysE inhibitory activity as previously reported (Verma et al., 2020). As shown in Supplementary Figure S4, quercetin at 100 μM exhibited moderate growth reduction of *E. coli*, in a similar extent to that induced by OGA at 50 μM. Under the conditions, OGA treatment reduced intrabacterial concentrations of cysteine, GSH, GSSH, and H_2_S. Quercetin treatment reduced intrabacterial levels of H_2_S. However, quercetin treatment did not reduce intrabacterial levels of cysteine and GSSH, and it rather increased intrabacterial levels of GSH. Although further experiments are needed, insufficient action of quercetin against bacterial cysteine reduction may be due to limited incorporation of quercetin by bacteria, as seen for gallic acid.

Since alterations of the cysteine biosynthesis by OGA can interfere with the redox homeostasis in *E. coli*, depleted cellular GSH levels could probably not only result from reduced GSH production due to cysteine limitation but also from GSH depletion by ROS-mediated oxidation of GSH to GSSG. In order to study whether OGA treatment affected GSH and GSSG levels in bacteria, we determined the intrabacterial levels of GSSG with or without OGA treatment by means of LC–MS/MS. As shown in Supplementary Figure S5, OGA treatment resulted in reduction of both GSH and GSSG, and hence, total of GSH and GSSG in bacteria. The ratios of GSH/GSSG were not significantly affected by OGA treatment under current experimental condition. These data suggest that reduction of GSH (and GSSH) by OGA treatment may be largely due to cysteine limitation. Considering these data together, our data here is the first to show that the small molecule inhibitor of CysE may reduce intracellular levels of cysteine and related molecules in bacteria.

As mentioned earlier, CysE catalyzes the conversion of serine to OAS. We thus hypothesized that blockage of CysE may lead to accumulation of the substrate serine. We measured the amino acid levels in wild-type *E. coli* treated with OGA treatment or untreated, and in the *cysE* mutant, by means of LC–MS/MS with the pre-column derivatization method. As expected, l-serine levels tend to increase in OGA-treated wild-type *E. coli*, and increased in the *cysE* mutant compared with untreated wild-type *E. coli* (Figures 3B,C and Supplementary Figures S6, S7). As an interesting result, OGA treatment and *cysE* deletion induced systemic changes in amino acid levels in bacteria (Figures 3B,C and Supplementary Figure S6). In OGA-treated *E. coli*, compared with untreated *E. coli*, levels of three amino acids (lysine, serine, and aspartic acid) increased or tend to increase, whereas levels of the other 17 amino acids decreased or tend to decrease. Such an alteration in the amino acid profile is similar to that in the *cysE* mutant except for glutamic acid, glycine, and glutamine. These findings provide additional support for the belief that OGA inhibits CysE in bacteria.

### 3.4. OGA treatment is used to sensitize bacteria to oxidative stress via accumulation of reactive oxygen species

**Figure 4:**
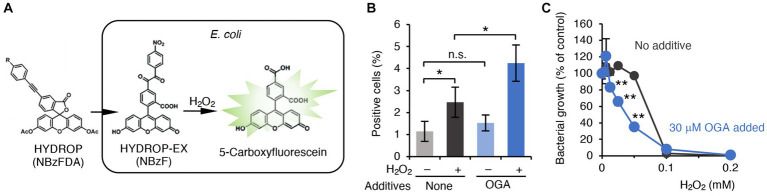
OGA-dependent augmentation of ROS production in C.I *E. coli*-1. **(A)** Schematic representation of H_2_O_2_ detection in bacteria. The acetylated form of the H_2_O_2_ reactive fluorophore HYDROP was loaded with bacteria. After hydrolytic removal of acetyl groups, HYDROP can be oxidized with H_2_O_2_ to become fluorescent. The fluorescence intensity of the bacteria was determined by means of flow cytometry. **(B)** Percentage of fluorescence-positive cells under various conditions. Clinical isolates of *E. coli* were cultured in the presence or absence of 30 μM OGA for 4 h. After incubation for 90 min with 10 μM HYDROP, bacteria were treated with 1 mM H_2_O_2_ for 30 min or were untreated. The fluorescence intensity of the bacteria was determined by means of the BD FACSCalibur flow cytometer (BD Biosciences). Data are means ± SD (*n* = 3). *p < 0.05; **p < 0.01; n.s., not significant. **(C)** H_2_O_2_-induced bacterial killing. *E. coli* were cultured in the presence of the indicated concentrations of H_2_O_2_ with or without 30 μM OGA for 24 h. Data are means ± SD (*n* = 3). *p < 0.05; **p < 0.01; n.s., not significant.

GSH is a cysteine-containing tripeptide that acts as an antioxidant to protect organisms from oxidative stress caused by reactive oxygen species (ROS). Recent studies have also suggested that GSSH can behave as a more potent antioxidant than the parental GSH (Ida et al., 2014; Sawa et al., 2020). As shown earlier, OGA treatment reduced both GSH and GSSH levels in bacteria (Figure 3A). In addition, the *cysE* mutant was reportedly more vulnerable to hydrogen peroxide (H_2_O_2_)-induced bacterial killing (Italiano et al., 2021). We therefore questioned whether OGA treatment could sensitize bacteria to ROS-mediated bacterial killing via reduction of the antioxidant molecules GSH and GSSH. C.I *E. coli*-1 bacteria treated with OGA (30 μM) or untreated were loaded with the H_2_O_2_-sensitive fluorescent probe HYDROP (Figure 4A). Fluorescence intensities were then quantitated by means of flow cytometry (Supplementary Figure S8). Exposure to H_2_O_2_ increased the percentage of fluorescence-positive bacteria, which suggests that H_2_O_2_ penetrated bacteria and reacted with the deacetylated form of HYDROP (Figure 4B). OGA treatment alone did not affect the percentage of fluorescence-positive bacteria. OGA treatment, however, significantly increased the percentage of fluorescence-positive bacteria after H_2_O_2_ exposure compared with the percentage without OGA treatment (Figure 4B). Furthermore, it was found that OGA-treated C.I *E.coli*-1 became more vulnerable to H_2_O_2_-induced bacterial killing (Figure 4C). We used *katG* mutant and its wild-type counterpart, BW25113, as control experiment. The *katG* mutant lacks catalase gene, and hence, is more sensitive against H_2_O_2_-induced oxidative stress. As shown in Supplementary Figure S9, increase of fluorescent intensity upon H_2_O_2_ treatment was more obvious for *katG* mutant than for BW25113. In addition, *katG* mutant was more vulnerable against H_2_O_2_-induced bacterial killing than BW25113. Taken together, it was suggest that OGA treatment weakened the antioxidant capacities in *E. coli*.

### 3.5. OGA treatment is used to enhance antibiotic susceptibilities of multidrug-resistant Gram-negative bacteria

**Figure 5:**
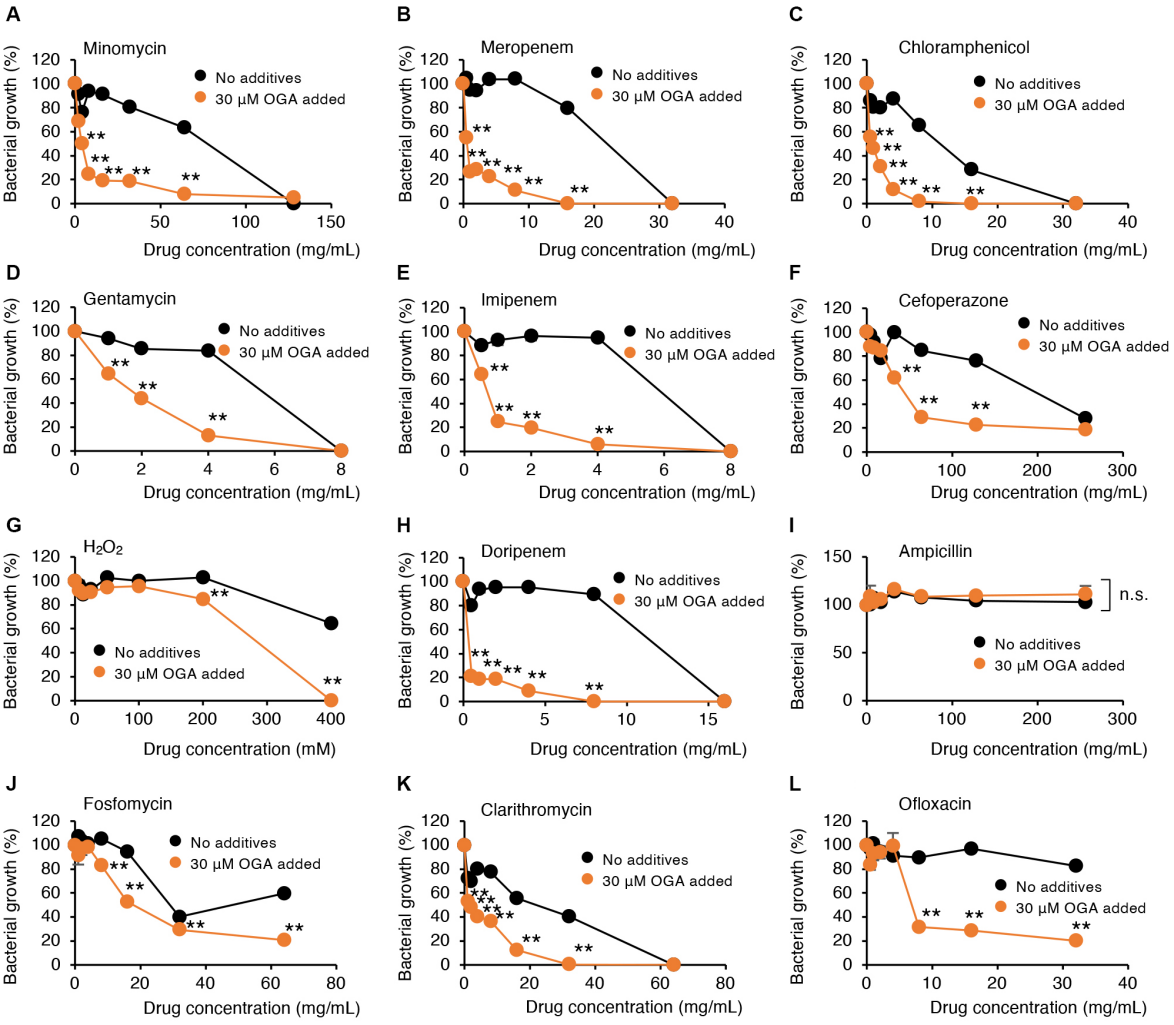
Modulation by OGA of antibiotic susceptibilities of C.I E.coli-2. Bacteria were cultured with or without OGA in the presence of the indicated concentrations of antibiotics. **(A)**, Minomycin; **(B)**, Meropenem; **(C)**, Chloramphenicol; **(D)**, Gentamycin; **(E)**, Imipenem; **(F)**, Cefoperazone; **(G)**, H_2_O_2_; **(H)**, Doripenem; **(I)**, Ampicillin; **(J)**, Fosfomycin; **(K)**, Clarithromycin; **(L)**, Ofloxacin. Data are means ± SD (*n* = 3). *p < 0.05; **p < 0.01; n.s., not significant.

**Figure 6:**
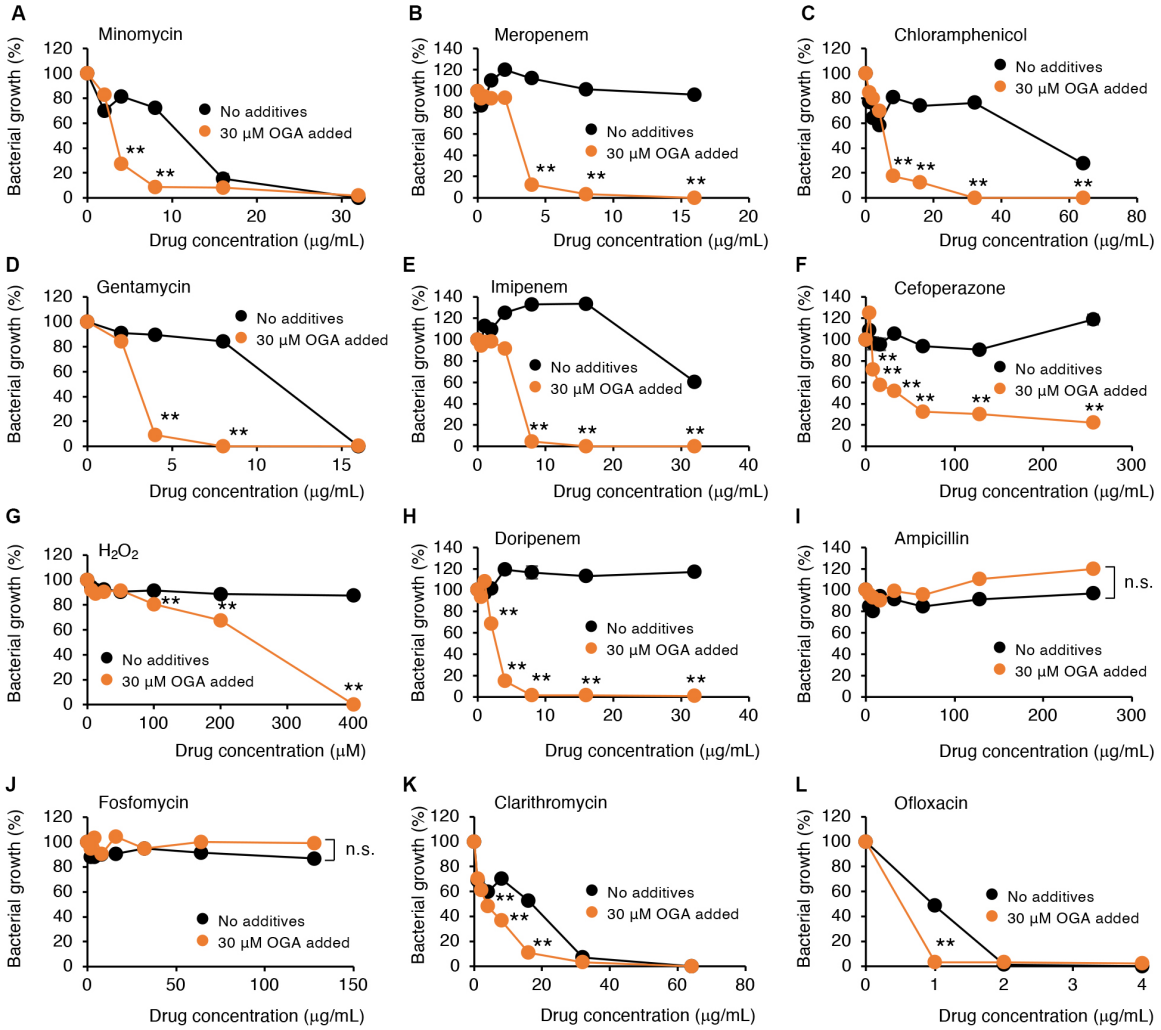
Modulation by OGA of antibiotic susceptibilities of C.I K. pneumoniae. Bacteria were cultured with or without OGA in the presence of the indicated concentrations of antibiotics. **(A)**, Minomycin; **(B)**, Meropenem; **(C)**, Chloramphenicol; **(D)**, Gentamycin; **(E)**, Imipenem; **(F)**, Cefoperazone; **(G)**, H_2_O_2_; **(H)**, Doripenem; **(I)**, Ampicillin; **(J)**, Fosfomycin; **(K)**, Clarithromycin; **(L)**, Ofloxacin. Data are means ± SD (*n* = 3). *p < 0.05; **p < 0.01; n.s., not significant.

Alkyl gallates, including OGA, reportedly enhanced antibiotic sensitivities in MRSA (Shibata et al., 2005; Tamang et al., 2022). In those studies, such enhancing effects of alkyl gallates were obvious for β-lactam antibiotics such as penicillin, ampicillin, and oxacillin. No report was available, however, for the effects of alkyl gallates on antibiotic susceptibility of drug-resistant Gram-negative bacteria. We therefore studied whether OGA treatment affects the antibiotic susceptibility of multidrug-resistant Gram-negative bacteria. We used two bacterial strains, C.I *E. coli*-2 and C.I *K. pneumoniae*, both of which were isolated from hospitalized patients. Conventional antibiotic sensitivity tests suggested that those bacteria showed resistance to many types of antimicrobial agents, particularly β-lactam antibiotics (Supplementary Tables S1, S2). As for wild-type *E. coli*, OGA treatment sensitized those clinical isolates to H_2_O_2_-induced bacterial killing (Figures 5G, 6G). As summarized in Table 1, OGA treatment reduced the minimum inhibitory concentration (MIC) values for carbapenem-type antibiotics two-fold in C.I *E. coli*-2 and more than four-fold in C.I *K. pneumoniae*. As Supplementary Tables S1, S2 indicate, the susceptibilities of those bacteria against carbapenems were enhanced in the presence of dipicolinic acid (DPA), which suggests that those bacteria may express metallo-β-lactamases (Kimura et al., 2005). To confirm this, we used PCR to detect IMP-1, the most prevalent type of metallo-β-lactamase in Japan (Nishio et al., 2004). As seen in Supplementary Figure S10, PCR bands were clearly detected in both clinical isolates. These data suggest that OGA may increase the antibiotic activity of carbapenems even against Gram-negative bacteria expressing metallo-β-lactamases.

**Table 1:** Effects of octyl gallate on MIC values of multidrug resistant *E. coli* and *K. pneumoniae.*

Bacteria	C.I *E. coli*-2		C.I *K. pneumoniae*	
Drugs	MIC (μg/mL)		MIC (μg/mL)	
	OGA (−)	OGA (+)	OGA (−)	OGA (+)
Ampicillin	>256	>256	>256	>256
Cefoperazone	>256	>256*	>256	>256*
Meropenem	32	16	>16	8
Imipenem	16	8	>32	8
Doripenem	16	8	>32	8
Ofloxacin	>32	>32*	2	1
Clarithromycin	64	32	64	32
Fosfomycin	>64	>64	>128	>128
Gentamycin	8	8	16	8
Minomycin	128	128*	32	32*
Chloramphenicol	16	8	>64	32
H_2_O_2_ (mM)	>13.6	13.6	>13.6	13.6

*Although clear MIC was not observed, bacterial growth was largely suppressed under combined treatment with OGA. Clinical isolates were cultured in the absence or the presence of 30 μM OGA in M9 + Glc + CA medium at 37°C for 24 h. Bacterial growth was determined by turbidity measured at 655 nm.

In contrast to reported effects against MRSA, OGA treatment did not sensitize those bacteria to ampicillin. Although the extent of enhancement was not very high (~2-fold), OGA treatment sensitized those clinical isolates of bacteria to different types of antimicrobial agents including ofloxacin (quinolone), clarithromycin (macrolide), gentamycin (aminoglycoside), minomycin (tetracycline), and chloramphenicol (Figures 5, 6 and Table 1).

### 3.6. Structural analyses

To gain insight into how gallate derivatives inhibit CysE, we performed structural analyses to determine the possible binding sites of gallate derivatives to CysE and their influence on enzymatic reactions. The overall structure of StCysE and its cysteine-binding site are described in Supplementary results and Supplementary Figures S11, S12.

#### 3.6.1. CoA-binding site of StCysE

**Figure 7:**
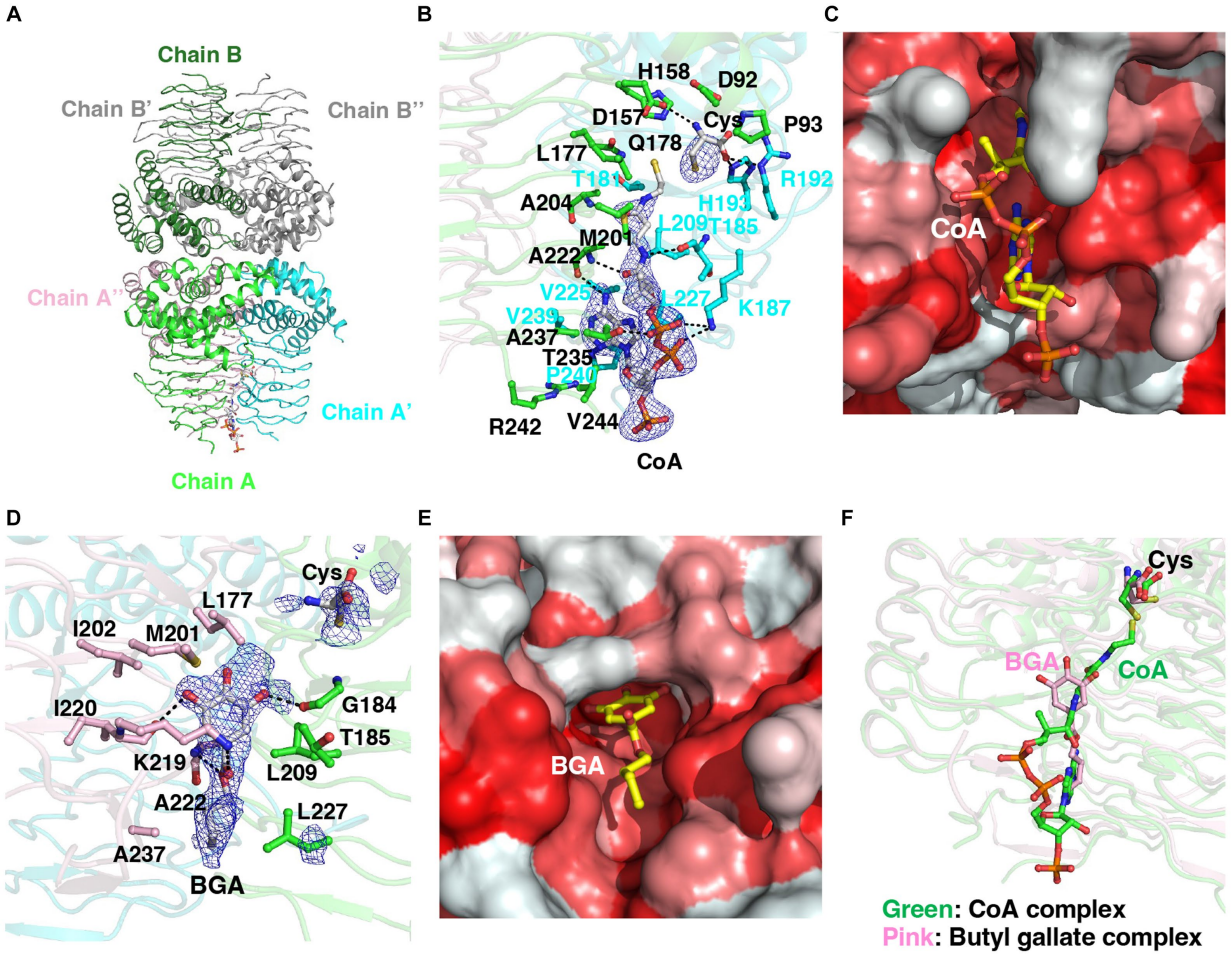
Structural analyses of the StCysE-CoA complex and BGA-binding site. **(A)** The crystal packing of the StCysE-CoA complex. Two subunits made up the asymmetric unit. Each subunit is on a crystallographic three-fold axis, and together they form the trimer. **(B)** The binding mode of CoA with an electron density map. The residues around 4 Å from the CoA molecule are shown as stick models. The mesh map is contoured with 2.0 sigma. **(C)** The CoA molecule binds in the hydrophobic cavity between subunits. Colors are according to the hydrophobicity scale: red, high hydrophobicity; white, low hydrophobicity. **(D)** The binding mode of BGA with an electron density map. The residues around 4 Å in the BGA molecule are shown as stick models. The mesh map is contoured with 1.5 sigma. **(E)** BGA binds in the hydrophobic cavity between subunits. Colors are according to the hydrophobicity scale: red, high hydrophobicity; white, low hydrophobicity. **(F)** The binding site of BGA almost overlaps that of CoA. BGA and CoA molecules are shown in pink and green, respectively.

We determined the crystal structure of a complex of StCysE and CoA. Two subunits of the StCysE-CoA complex were in an asymmetric unit: one subunit formed a trimer that was related to a crystallographic three-fold axis, and the other subunit also formed a trimer that was related to the same crystallographic three-fold axis; together they formed a hexamer (Figures 7A,B: residues: 1–245). The CoA molecule bound to a hydrophobic crevice located between β-helices at three equidistant active site locations related to a three-fold axis near a cysteine-binding site (Figures 7B,C). The di-phosphate portion of CoA interacted with the side chain of Lys-187 via electrostatic interaction and formed a hydrogen bond to the side chain of Thr-235. The pantothenic group of CoA interacted with the main chain of Thr-185 and Ala-222. The adenine group of CoA was fit in the hydrophobic pocket of the StCysE (Figure 7C). There was no electron density in the C-terminal region of StCysE (residues: 245-C-terminus), so the hydrophobic region of StCysE was exposed to the solvent.

#### 3.6.2. Binding site of butyl gallate to StCysE

Because of the poor water solubility of OGA, we could not crystalize StCysE in a complex with OGA. We did, however, successfully obtain a co-crystal of StCysE complexed with BGA and hence could determine its crystal structure. The C-terminal region of the BGA-StCysE complex (residues: 248-C-terminus) is not electron dense. BGA binds to the hydrophobic pocket of StCysE where it is very close to the binding site of CoA (Figures 7D,E). The hydroxyl group of BGA interacts with the main chain O atom of Gly-184 and the main chain O atom of Ile-220. The ketone group of BGA interacts with the side chain of Lys-219. The ester O atom of BGA interacts with the main chain N atom of Ala-222. The hydrophobic alkyl chain of BGA is surrounded by hydrophobic residues of the enzyme: Leu-209, Ala-222, Leu-227, and Ala-237 (Figures 7D,E). The binding site of acetyl-CoA overlaps with the BGA-binding site (Figure 7F). Thus, binding of BGA to the enzyme may compete with binding of acetyl-CoA, which would result in inhibition of StCysE activity. It should be note that all these amino acids that make up the binding site of butyl gallate are conserved between StCysE and EcCysE (Supplementary Figure S13).

### 3.7. Enzyme kinetics study

Structural analysis suggests that BGA can bind to CysE and inhibit enzyme activity. To investigate this possibility, we determined the kinetic parameters of CysE in the presence of BGA. We tested the catalytic rate of CysE at different concentrations of acetyl-CoA (0, 0.1, 0.5, 1, 3, and 5 mM) in the absence or presence of BGA at concentrations of 25 and 50 μM. The higher BGA concentration increased both the apparent Michaelis–Menten constant and the apparent maximum rate (Supplementary Figure S14). This result suggests that BGA inhibits the CysE reaction in a mixed fashion against acetyl-CoA. As discussed above, BGA can bind to CysE close to the acetyl-CoA-binding site (Figure 7F), which may result in competitive inhibition of the enzyme. Our data suggested that BGA may also bind to the CysE-acetyl-CoA complex and thus reduce the enzyme activity. The binding site of BGA to the CysE-acetyl-CoA complex warrants additional investigation.

## 4. Discussion

This study determined, for the first time, that alkyl gallates could act as potent inhibitors of bacterial CysE *in vitro* and in bacteria. Alkyl gallates have been reported to exhibit antibacterial activities against some bacteria and fungi, and the chain lengths of alkyl gallates have been demonstrated to differentially affect the antibacterial activities. Among alkyl gallates studied, OGA exhibited the strongest antimicrobial activities against *Salmonella Choleraesuis* (Kubo et al., 2002), *Bacillus subtilis* (Kubo et al., 2004), *Saccharomyces cerevisiae*, *Zygosaccharomyces bailii*, *Monilia albicans*, and *Aspergillus niger* (Kubo et al., 2001). In consistent with those studies, we observed that OGA exhibited superior antibacterial activities compared with gallic acid and shorter alkyl chain derivatives under the current experimental settings. Our study suggested that introduction of alkyl chain into gallic acid moiety promoted intrabacterial uptake (Supplementary Figure S3).

Multiple modes of action have been suggested for antibacterial effects of OGA. Shi et al. (2021) and Zhang et al. (2021) observed that OGA could perturbate integrity of membrane lipid bilayer, that subsequently activate tricarboxylic acid cycle to over-produce NADH. Excess NADH thus formed induces unregulated electron transport chain activation, leading to the production of superoxide. In this process, metabolic pathways of urea cycle, purine metabolism, and amino acids are also modulated. In those studies, elevation of certain amino acids, including valine, arginine, alanine, proline, and glutamate was observed when *Pseudomonas fluorescens* was treated with OGA in nutrient rich LB medium. However, such alterations of amino acids were not observed for OGA-treated *E. coli* (Figure 3). Different experimental conditions such as bacteria species and nutrient status may contribute to the observed discrepancy. On the other hand, we observed that similar alteration of amino acid profiles in both OGA treatment of *E. coli* as well as in *cysE* mutant when those bacteria were cultured in nutrient limited M9 medium (Figure 3). This finding may further support the notion that OGA treatment blocked cysteine biosynthesis as in *cysE* mutant, resulting in modulation of systemic amino acid metabolisms. We also observed that levels of certain amino acids differently modulated in OGA-treated bacteria and in *cysE* mutant including glutamic acid, glycine, and glutamine (Figure 3). Mechanism involved in such difference is currently unknown. Such alterations may be due to OGA treatment independent of CysE inhibition, and thus, warrants further investigation.

The bacteria, named the ESKAPE (*Enterococcus faecium*, *S. aureus*, *K. pneumoniae*, *Acinetobacter baumannii*, *Pseudomonas aeruginosa*, and *Enterobacter* species) bacteria, have been considered to account for the majority of nosocomial infections worldwide, with an increasing incidence of drug resistance every year (Boucher et al., 2009; Rice, 2010). Antimicrobial adjuvants that enhance the activity of drugs and can minimize microbial resistance has attracted much attention as an approach to strengthen the efficacy of existing drugs for the treatment of those ESKAPE bacteria (Wright, 2016; Domalaon et al., 2018; Dubey et al., 2020). Although CysE inhibitors have been considered to become potential antimicrobial adjuvants (Hicks et al., 2022), no reports are yet available showing CysE inhibitors could sensitize antibiotic responses to Gram-negative bacteria. As shown in this study, OGA could intensify the susceptibilities of the ESKAPE bacteria, including *E. coli* and *K. pneumoniae*, to carbapenems such as doripenem, meropenem, and imipenem, suggesting that CysE inhibitors may show promise as novel adjuvants to enhance antibiotic activity of carbapenems.

OGA is already approved as a food additive by Food and Drug Administration in the USA and its safety and manufacturing methods have been established. Qiu et al., reported that OGA could be taken from edible oils and oil emulsions amounting to 100 mg/kg supporting that OGA is low toxicity to human health (Qiu et al., 2022). This suggests that market-targeted dugs including OGA have good advantages for going to clinical practice smoothly. In addition, we showed in this study that OGA at 30 μM could sensitize multidrug resistant Gram-negative bacteria against various types of antibiotics (Figures 5, 6). Tamang et al., also reported that OGA strongly potentiated antibiotic activities of diverse antibiotics for MRSA (Tamang et al., 2022). It should be noted that even higher concentration of OGA (~100 μM) was not toxic to human normal cell line (MCF-10A) (Sales et al., 2018). We obtained similar result that OGA at 100 μM only slightly reduced cell viability of human embryonic kidney cell line HEK293T cells (Supplementary Figure S15). These data suggest that OGA can be used as an antibiotic adjuvant to potentiate existing antibiotics against both Gram-negative and Gram-positive bacteria.

Certain bacteria can produce cysteine from L-homocysteine via intermediate formation of cystathionine, called reverse transsulfuration pathway (Guedon and Vestraete, 2006; Paritala and Carroll, 2013). Blockage of CysE-CysK dependent cysteine biosynthesis may activate bacterial utilization of the reverse transsulfuration pathway, leading to the potential resistance against CysE inhibitors. In the reverse transsulfuration pathway, cystathionine γ-lyase (CSE) is the last enzyme to produce cysteine (Guedon and Vestraete, 2006; Paritala and Carroll, 2013). Shatalin et al., reported that indole-based compounds such as NL1 selectively inhibited bacterial CSE but not so much for human CSE (Shatalin et al., 2021). They also showed that NL1 and its related compounds sensitized *S. aureus* and *P. aeruginosa* against antibiotics. As shown in Supplementary Figure S1, cysteine is also produced from serine through intermediate formation of *S*-sulfo-L-cysteine in some bacteria such as *Mycobacterium tuberclosis* (Agren et al., 2008). This pathway is catalyzed by CysM (also known as CysK2). Furthermore, inhibitors of CysM/CysK2 exhibited antibacterial activity against *M. tuberculosis*, indicating that blockage of cysteine biosynthetic pathway is promising approach to various pathogenic bacteria (Yogeeswari et al., 2016). Combination of inhibitors against CysE, CysM (CysK2) and CSE warrants further investigation to prevent potential occurrence of resistant bacteria by blocking both pathways.

In summary, CysE inhibitors may be used to treat infectious diseases caused by drug-resistant Gram-negative bacteria not only via direct antibacterial activity but also by enhancing therapeutic potentials of existing antibiotics. Additional study is needed, however, to clarify the therapeutic effects of CysE inhibitors *in vivo*.

## 5. Conclusion

This study determined, for the first time, that alkyl gallates could act as potent inhibitors of bacterial CysE *in vitro* and in bacteria. Detailed analyses using OGA as a representative alkyl gallate model indicated that OGA treatment markedly reduced intrabacterial levels of cysteine and its related metabolites including GSH, GSSH, and H_2_S to levels comparable to those of the CysE deletion mutant. OGA treatment increased intracellular ROS levels after H_2_O_2_ exposure, and more important, sensitized *E. coli* to H_2_O_2_-mediated bacterial killing. Furthermore, OGA treatment intensified the susceptibilities of metallo-β-lactamase-expressing Gram-negative bacteria (*E. coli* and *K. pneumoniae*) to carbapenems such as doripenem, meropenem, and imipenem. Structural and kinetic analyses demonstrated that alkyl gallates may compete with acetyl-CoA, a co-substrate of CysE reaction, when binding to the enzyme. These findings suggest that CysE inhibitors may show promise as novel adjuvants to enhance antibiotic activity of carbapenems. Additional study is needed, however, to clarify the therapeutic effects of CysE inhibitors *in vivo*.

## Data availability statement

The raw data supporting the conclusions of this article will be made available by the authors, without undue reservation.

## Author contributions

TT: Investigation, Methodology, Writing – original draft. KO: Formal analysis, Funding acquisition, Investigation, Writing – original draft. TSh: Methodology, Writing – original draft. KM: Investigation, Methodology, Writing – review & editing. TZ: Investigation, Writing – review & editing. HT: Investigation, Methodology, Writing – review & editing. TI: Methodology, Writing – review & editing. KaH: Methodology, Writing – review & editing. KoH: Investigation, Writing – review & editing. AR: Investigation, Writing – review & editing. LW: Investigation, Writing – review & editing. KY: Investigation, Resources, Writing – review & editing. MM: Resources, Writing – review & editing. KeH: Investigation, Methodology, Writing – review & editing. TN: Investigation, Methodology, Writing – original draft. TA: Investigation, Methodology, Writing – review & editing. TSa: Conceptualization, Funding acquisition, Supervision, Writing – original draft. YM: Methodology, Writing – review & editing.
