# Supplementary material for: Alkyl gallates inhibit serine O-acetyltransferase in bacteria and enhance susceptibility of drug-resistant Gram-negative bacteria to antibiotics
Source: Front Microbiol. 2023 Oct 27;14:1276447. doi: 10.3389/fmicb.2023.1276447 (PMC10641863; doi:10.3389/fmicb.2023.1276447)
Supplement: Supplementary file 1 [file Data_Sheet_1.pdf]

Supplementary Material

1 Supplementary Results

1.1 Overall structure of StCysE

We determined the structures of StCysE by means of the molecular replacement method and using the structure of *E. coli* CysE (EcCysE) (PDB code: 1T3D)[1] as a search model, and we established the final structures at 1.8–2.5 Å resolution (residues: 5-261). The asymmetric unit of the cysteine-complex of StCysE crystal is a trimer with a non-crystallographic three-fold axis. The trimer of the N-terminal domain interacts mainly via hydrophobic interactions to form a dimer of the trimer (i.e., a hexamer) related to a crystallographic two-fold axis (Supplementary Figure 11A). The overall structures of StCysE and EcCysE are very similar (root-mean-square deviation, or rmsd, of Cα atoms are 0.62 Å between the corresponding trimer), and the sequence identity between StCysE and EcCysE is 93% (Supplementary Figure 11B). The StCysE monomer is composed of two domains, the N-terminal domain (residues: 1-141) and the C-terminal domain (residues: 142-261), probably as EcCysE (Supplementary Figure 11C). Like EcCysE, the N-terminal domain is composed of eight α-helices (α1-α8) and the C-terminal domain forms a left-handed β-helix.

1.2 Cysteine-binding site of StCysE

In StCysE, the cysteine-binding site is located at the N-terminal side of the left-handed β-helix domain between the second and third β-strand repeats of two adjacent subunits (Supplementary Figure 12A; residues: 5-261). The amino group of the cysteine molecules interacts with the carboxyl groups of Asp-92 and Asp-157 through electrostatic interactions. The carboxyl groups of the cysteine molecule interact with Arg-192 of the adjacent subunit through electrostatic interactions. The carboxyl groups of the cysteine molecule interact with two water molecules through hydrogen bonds. The thiol group of the cysteine molecule is sandwiched with His-158 and His-193 of the adjacent subunit (Supplementary Figure 12B).

The amino group of the serine molecules interacts with the carboxyl groups of Asp-92 and Asp-157 through electrostatic interactions (residues: 5-261). The carboxyl groups of the serine molecule interact with Arg-192 of the adjacent subunit through electrostatic interactions. The carboxyl groups of the serine molecule interact with two water molecules through hydrogen bonds, just as for cysteine. The hydroxyl group of the serine molecule is sandwiched with His-158 and His-193 of the adjacent subunit (Supplementary Figure 12C). Thus, the serine molecule binds to StCysE in a manner similar to that of cysteine (Supplementary Figure 12D). These findings agree with data for CysE1 from *Entamoeba histolytica*.[2] The rmsd of the corresponding Cα atoms is 0.79 Å between Cys-StCysE and Ser-StCysE (Supplementary Figure 12D).

2 Supplementary Methods

2.1 Cytotoxicity assay.

HEK293T cells were seeded in 96-well plate at a density of 8 x 10^5 cells/mL. On the next day, the cells were treated by OGA in a serial concentration from 0 to 500 μM. After incubation for 8 hours, two-hundred microliter of MTT-containing DMEM was replaced into each well. Following incubation for 2 h, one-hundred and fifty microliter of culture supernatant was then removed from each well. The formed formazan crystals were dissolved by addition of 100 μL of

MTT stop solution (0.4% HCl, 10% Triton-X 100 in isopropanol). The absorbance was measured through a microplate reader (Bio-Rad Laboratories) at 490 nm, with 655 nm as the reference wavelength.

3 Supplementary References

1. Kumar S, Raj I, Nagpal I, Subbarao N, Gourinath S. (2011). Structural and biochemical studies of serine acetyltransferase reveal why the parasite Entamoeba histolytica cannot form a cysteine synthase complex. J Biol Chem. 286(14), 12533-41. doi: 10.1074/jbc.M110.197376.

2. Pye VE, Tingey AP, Robson RL, Moody PC. (2004). The structure and mechanism of serine acetyltransferase from Escherichia coli. J Biol Chem. 279(39), 40729-36. doi: 10.1074/jbc.M403751200.

4 Supplementary Figures

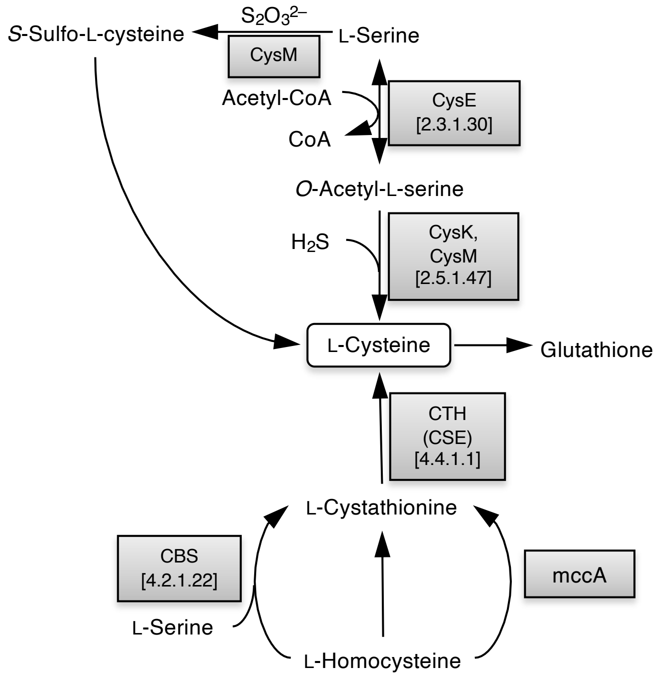

Supplementary Figure 1. L-Cysteine biosynthesis pathway. Acetyl-CoA, acetyl coenzyme A; CysE, serine *O*-acetyltransferase; CysK, *O*-acetylserine sulfhydrylase or cysteine synthase; CysM (CysK2), cysteine synthase; CBS, cystathionine β-synthase; CSE, cystathionine γ-lyase; CoA, coenzyme A; H_2S, hydrogen sulfide; mccA, *O*-acetylserine-dependent cystathionine β-synthase. Numbers in parenthesis are Enzyme Commission numbers.

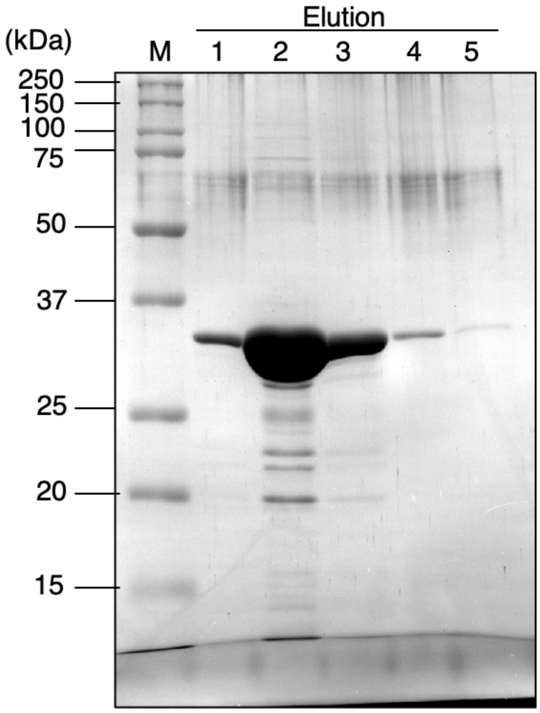

Supplementary Figure 2. Purification of CysE enzyme. Cells lysates of *E. coli* expressing *Salmonella* CysE was subjected to Ni-NTA agarose affinity chromatography purification. CysE was eluted by elution buffer containing 250 mM imidazole five times. Elution fractions were analyzed by sodium dodecyl sulfate-polyacrylamide gel electrophoresis with Coomassie Brilliant Blue staining.

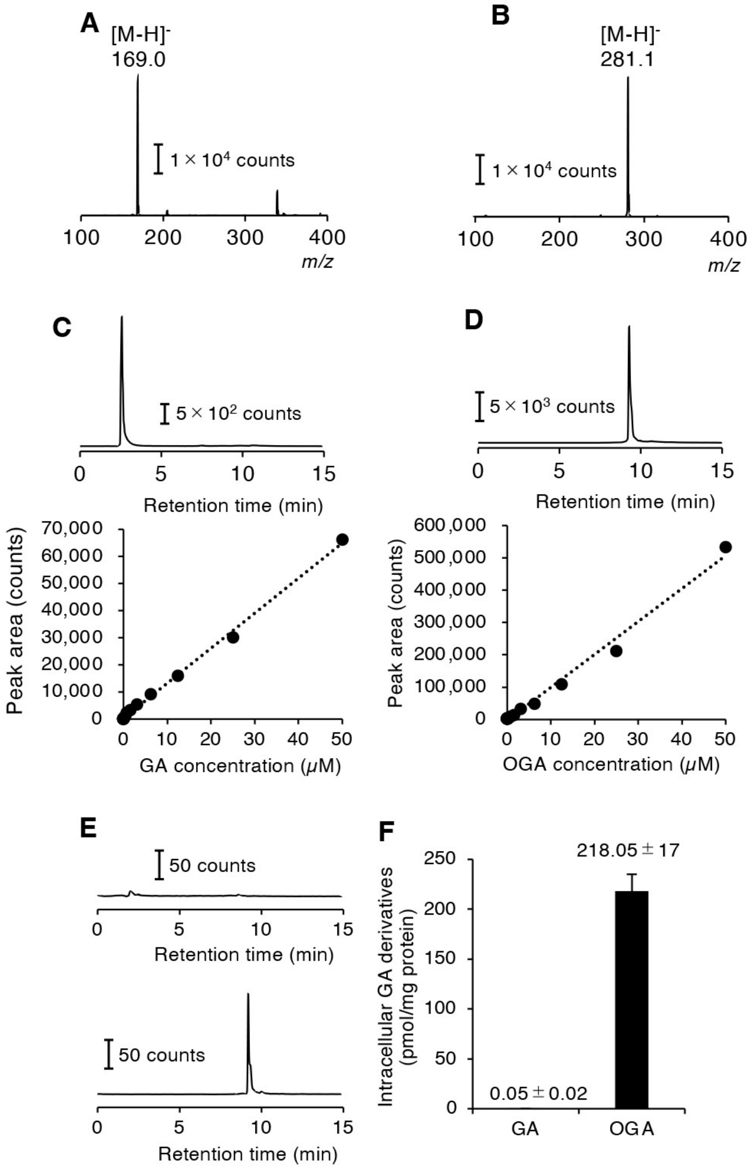

Supplementary Figure 3. Uptake of gallic acid and OGA by *E. coli*. Mass spectra of gallic acid (A) and octyl gallate (B). (C) Representative MRM chromatogram (upper panel) and calibration curve (lower panel) for GA. (D) Representative MRM chromatogram (upper panel) and calibration curve (lower panel) for OGA. (E) Representative MRM chromatogram detecting GA and OGA from *E. coli* BW25113 treated with 100 µM GA or OGA. Upper panel, chromatogram for GA, sample from *E. coli* treated with GA. Lower panel, chromatogram for OGA, sample from *E. coli* treated with OGA. *E. coli* bacteria were treated with 100 µM GA or OGA in M9+Glc+CA medium at 37 °C for 15 min. Cells were washed in PBS twice and then suspended in methanol. Samples were diluted 5 times with 0.1% formic acid and subjected to LC-MSMS. (F) Intracellular levels of GA derivatives from *E. coli* treated with GA or OGA. Data are means ± SD (n = 3).

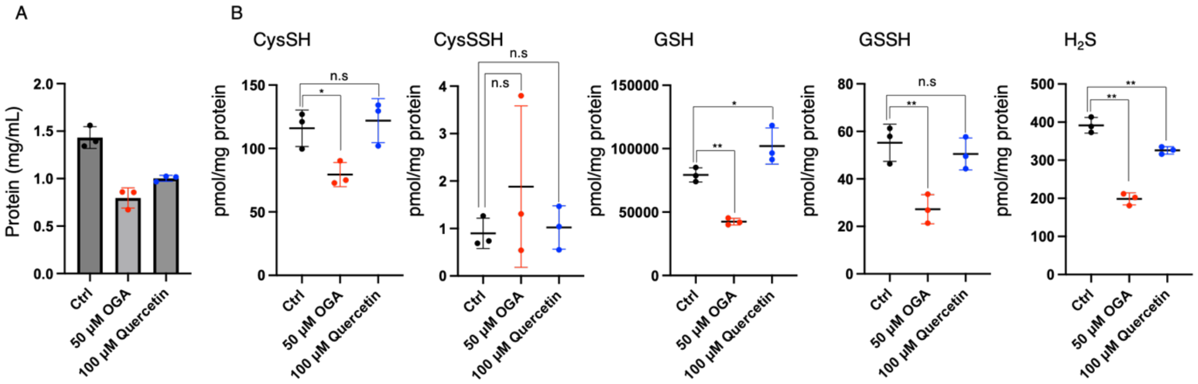

Supplementary Figure 4. Metabolomic analyses of *E.coli* treated with OGA or quercetin. *E.coli* BW25113 was treated with OGA (50 µM) (red), quercetin (100 µM) (blue) or untreated (black) were analyzed by using LC-MS/MS metabolomics with use of thiol-alkylating agent HPE-IAM. (A) Total protein levels of *E.coli* (black), *E.coli* treated with OGA (red) or *E.coli* treated with quercetin (blue). (B) Cysteine and its related molecules in *E.coli.* Data are means ± SD (n =3). *, $p < 0.05$; **, $p < 0.01$; n.s., not significant.

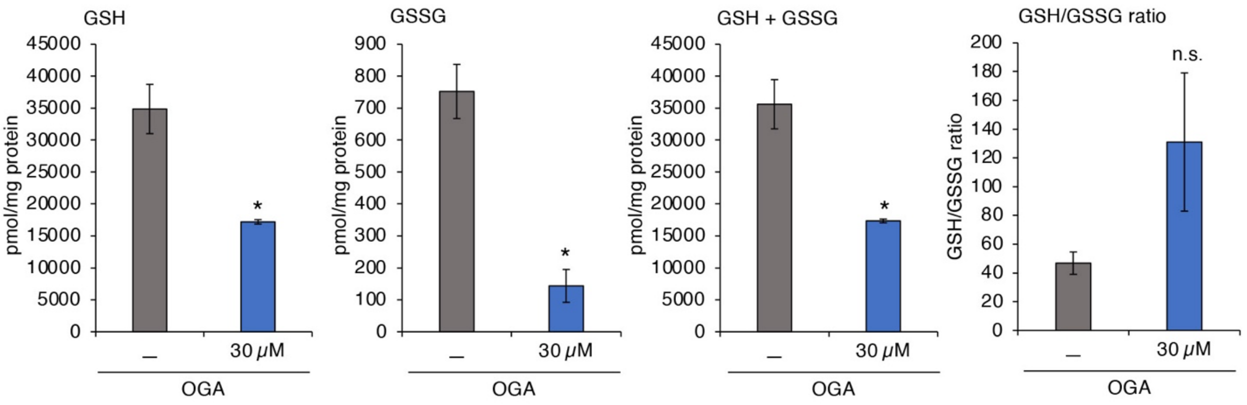

Supplementary Figure 5. Effects of OGA on intracellular GSH and GSSG in *E. coli*. *E. coli* BW25113 was treated by OGA at 30 μ M for 4 h, followed by subjected for measurements of GSH and GSSG. Data are means ± SD (n =3). *, $p < 0.05$; n.s., not significant.

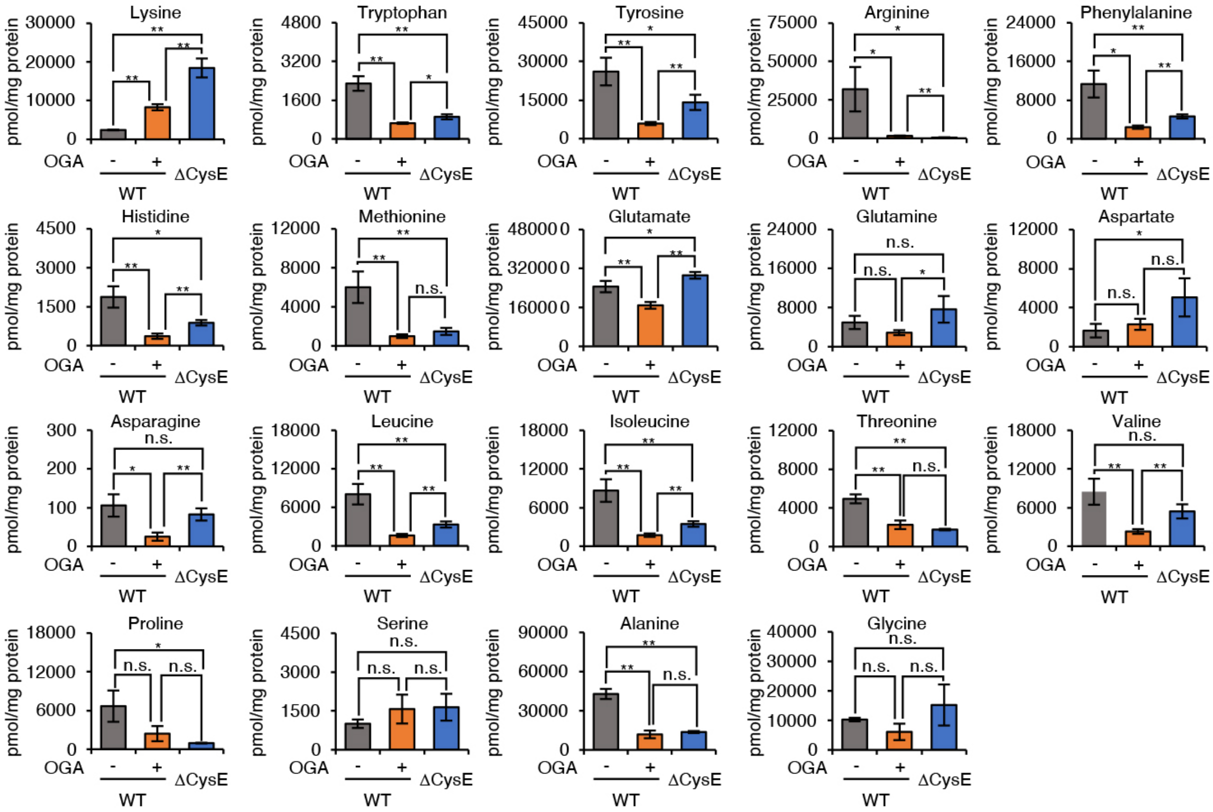

Supplementary Figure 6. Levels of amino acids in *E. coli*. Concentrations of amino acids were determined by using LC-MS/MS after APDS reaction. Data are means ± SD (n =3). *, $p < 0.05$; **, $p < 0.01$; n.s., not significant.

A

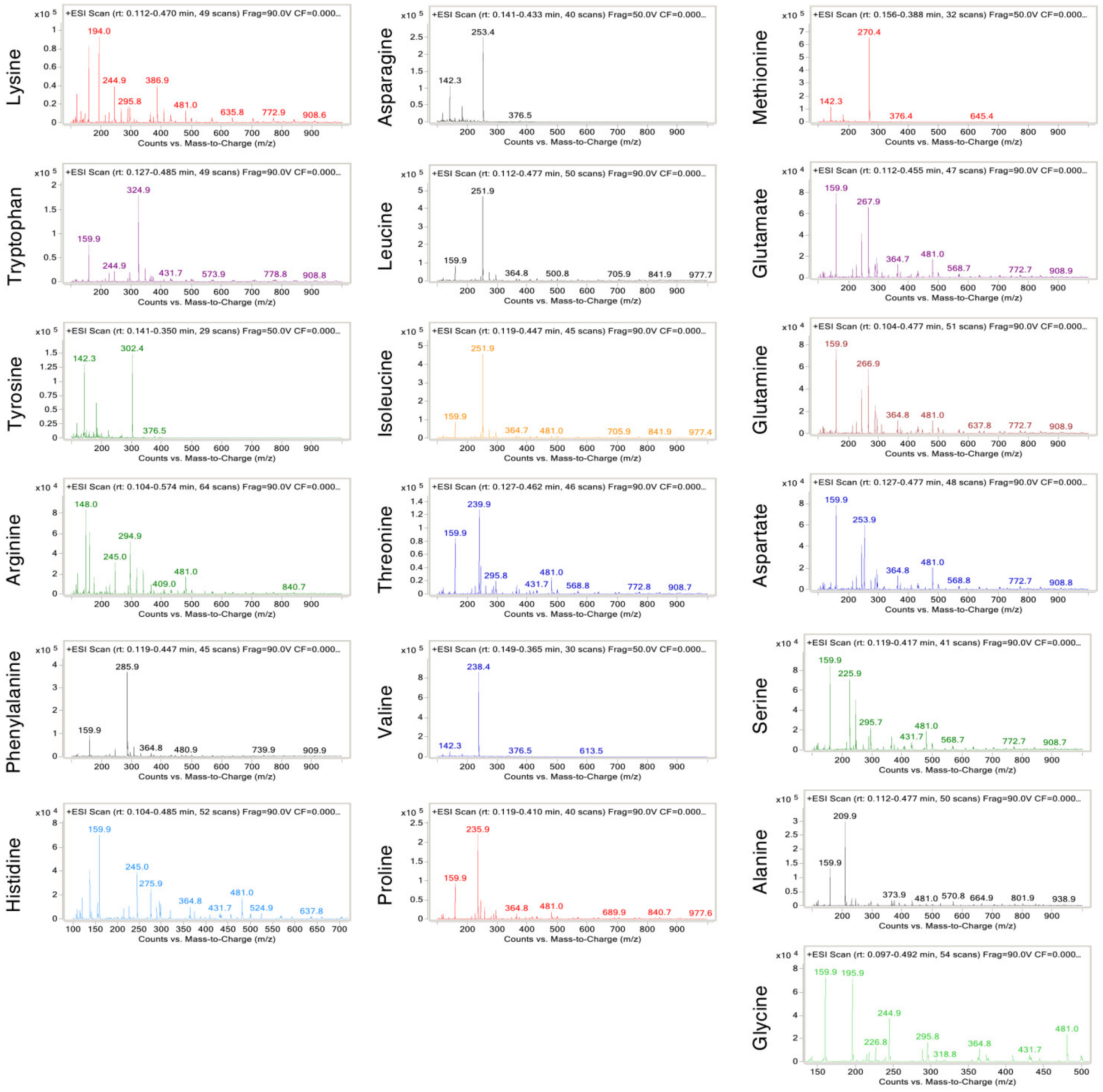
Lysine
Asparagine
Methionine
Tryptophan
Leucine
Glutamate
Tyrosine
Isoleucine
Glutamine
Arginine
Threonine
Aspartate
Phenylalanine
Valine
Serine
Histidine
Proline
Alanine
Glycine

B

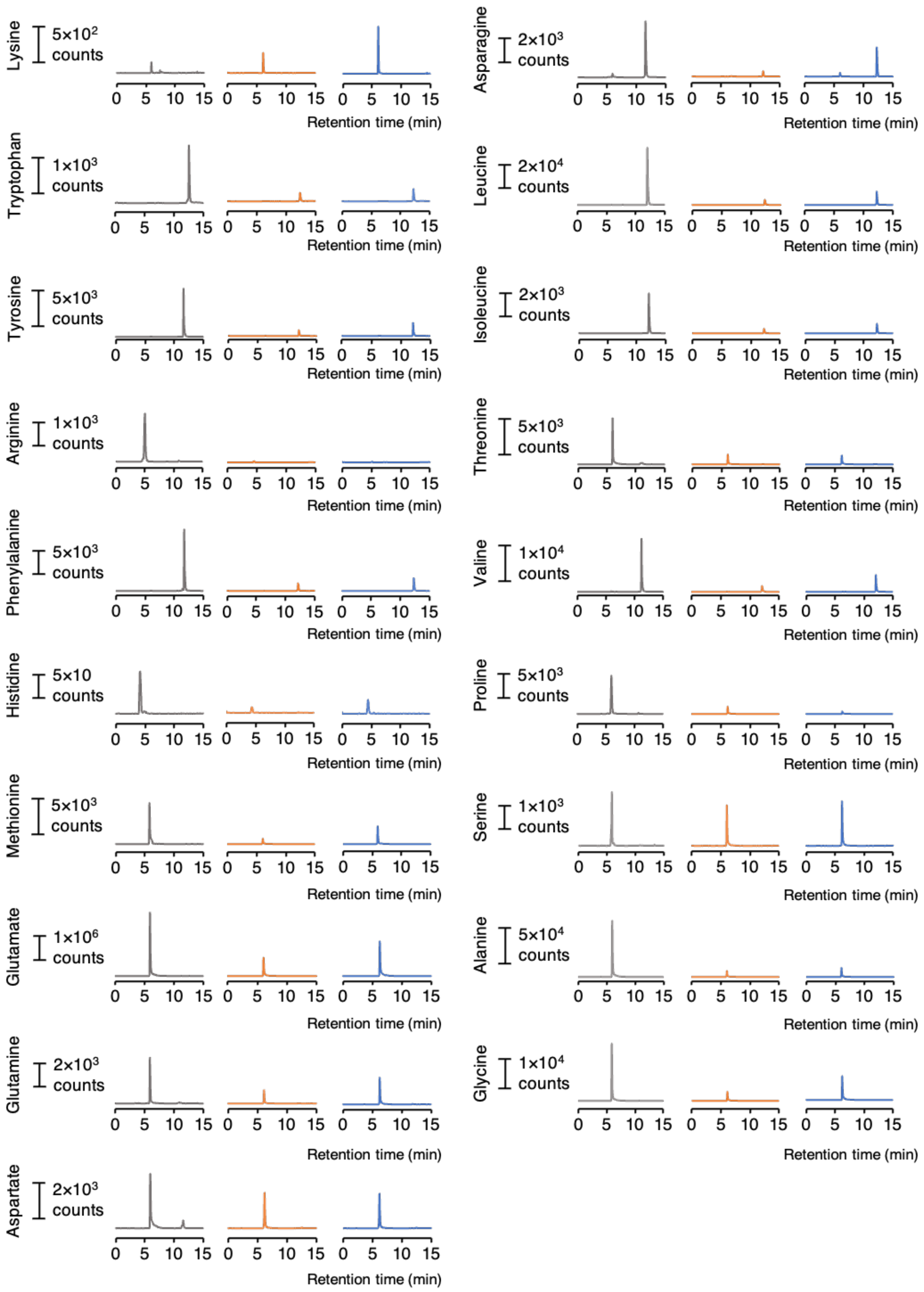

Supplementary Figure 7. Mass spectra (A) and MRM chromatograms (B) for APDS adducts of amino acids. Representive chromatograms of APDS adducts of amino acid detecting from wild-type *E. coli* (gray), wild-type *E. coli* treated with OGA (orange) and *cysE* deletion mutant (blue), respectively.

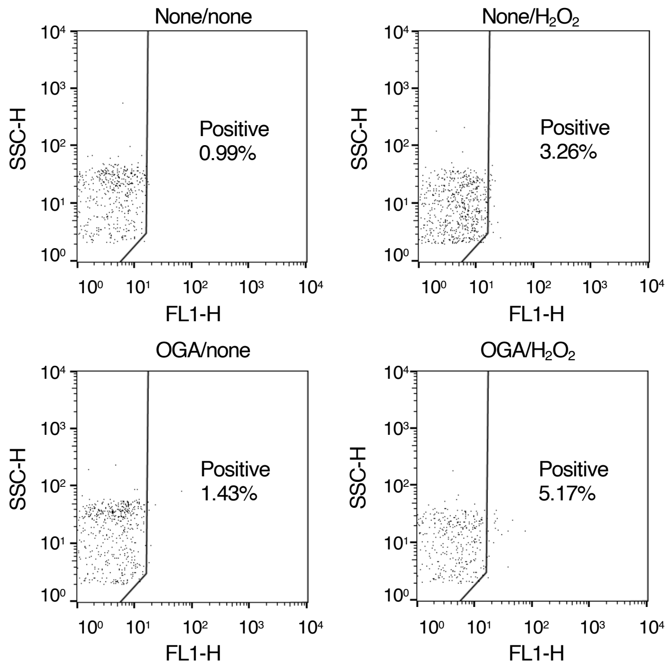

Supplementary Figure 8. Flow cytometric analyses of the effect of OGA on H_2O_2-treated clinical isolates of *E. coli*; related to Figure 4. C.I *E.coli*-1 was treated under various conditions as described in Figure 4. The number of fluorescence-positive cells was determined by means of a flow cytometer, and the dot plot data were processed by using FlowJo FACS analysis software (BD Biosciences). The bar graph in Figure 4 provides representative data.

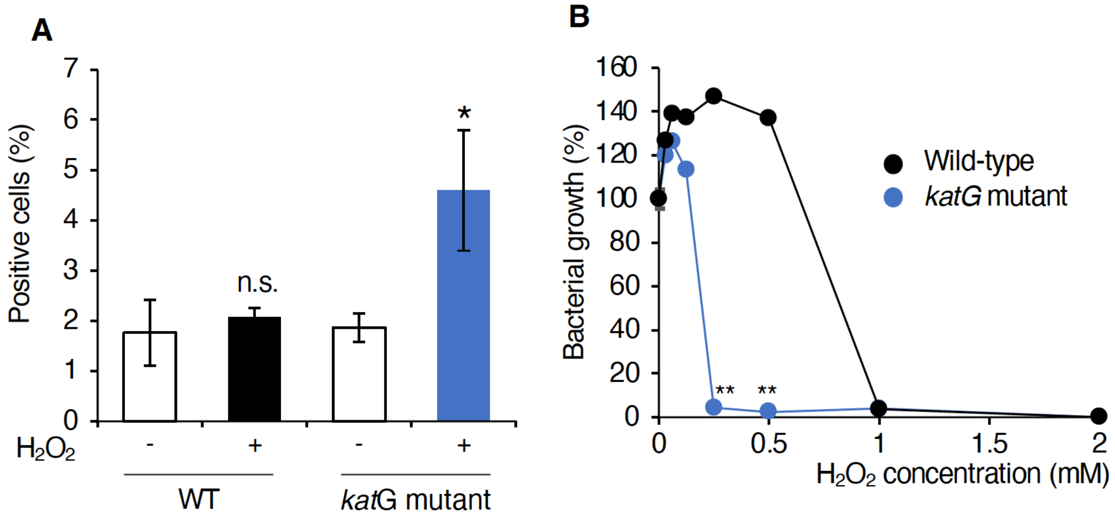

Supplementary Figure 9. Comparison of *katG* mutant and wild-type *E. coli* (BW25113) on intracellular ROS levels and sensitivity against H_2O_2-induced bacterial killing. (A) Percentage of fluorescence-positive cells with or without H_2O_2 treatment. Wild-type *E. coli* or *katG* mutant were incubated for 90 min with 10 µM HYDROP, followed by treating with 1 mM H_2O_2 for 30 min. The fluorescence intensity of the bacteria was determined by means of the BD FACSCalibur flow cytometer. (B) H_2O_2-induced bacterial killing. Bacteria were cultured in the presence of the indicated concentrations of H_2O_2 for 24 h. Bacterial growth was determined by measureing turbidity at 655 nm. Data are means ± SD (n =3). *, $p < 0.05$; **, $p < 0.01$; n.s., not significant.

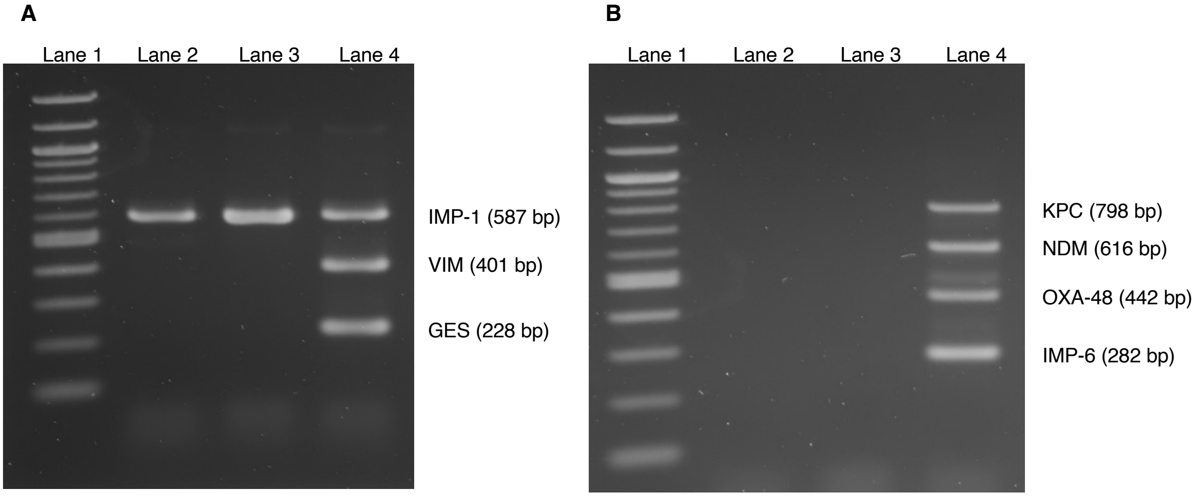

Supplementary Figure 10. PCR analysis of the expression of β-lactamases in bacteria used in this study. (A) IMP-1, VIM, and GES. Lane 1, molecular size marker; Lane 2, C.I *E.coli*-2; Lane 3, C.I *K. pneumoniae*; Lane 4, positive control. (B) KPC, NDM, OXA-48, and IMP-6. Lane 1, molecular size marker; Lane 2, C.I *E.coli*-2; Lane 3, C.I *K. pneumoniae*; Lane 4, positive control.

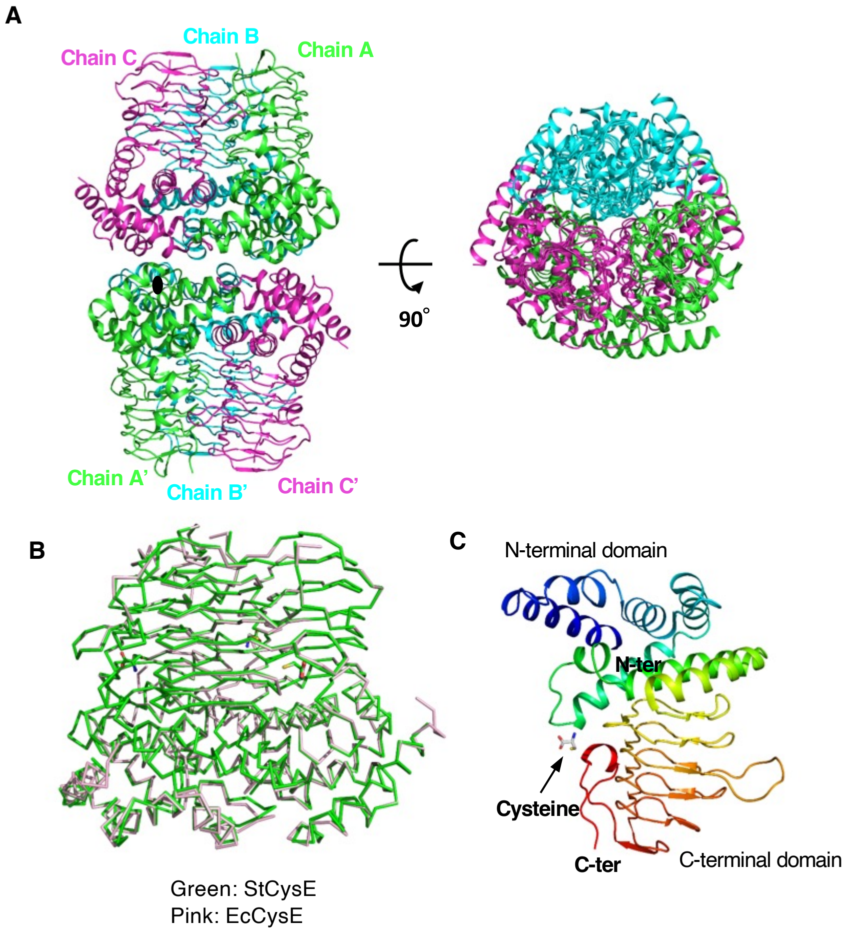

Supplementary Figure 11. Crystal structure of the StCysE. (A) Ribbon diagram of hexameric serine acetyltransferase (CysE). The upper trimer (chains A, B, and C) is related to a crystallographic two-fold axis (central ellipse), which forms the active hexamer. (B) Superimposed structures of the StCysE (green) and EcCysE (pink). (C) Monomer structure of StCysE in a rainbow color (N-terminus: blue, C-terminus: red). The cysteine molecule is shown as a white ball-and-stick model.

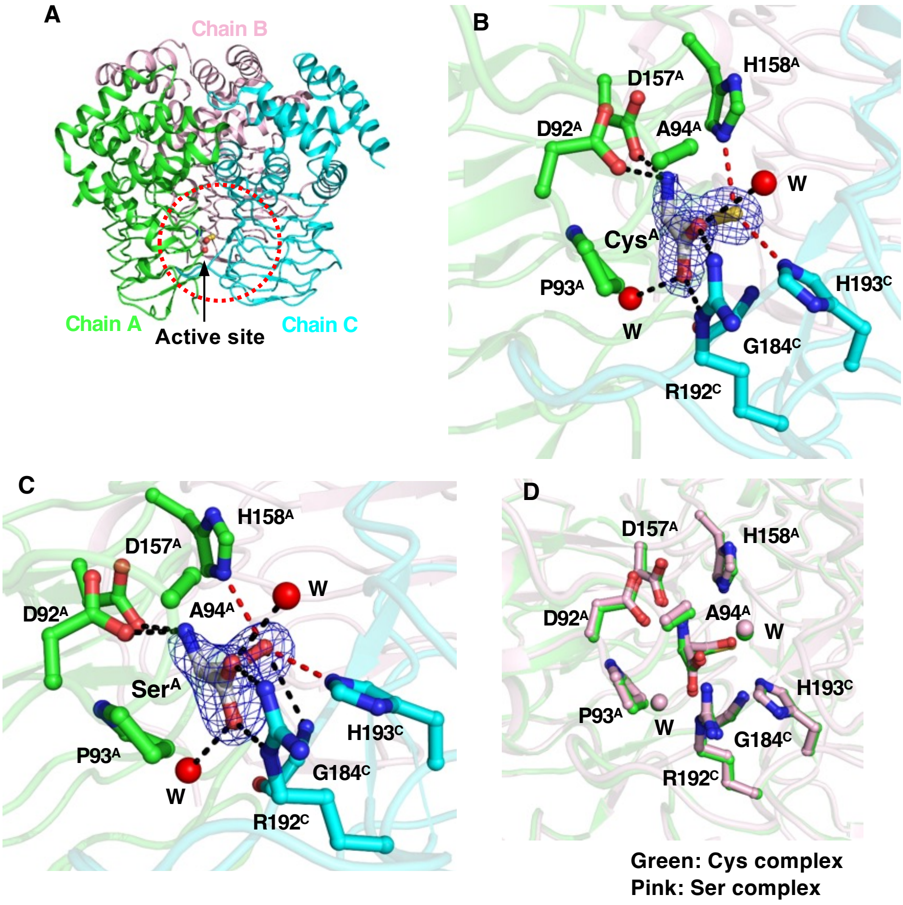

Supplementary Figure 12. (A) Ribbon diagram of trimer StCysE in a complex with cysteine. The active site is between subunits. (B) The binding mode of Cys with an electron density map. The residues around 4 Å from the cysteine molecule are shown as stick models. The mesh map is contoured with 2.0 sigma. (C) The binding mode of Ser with an electron density map. The residues around 4 Å from the serine molecule are shown as stick models. The mesh map is contoured with 2.0 sigma. Hydrogen bonds are shown as dashed lines, water molecules that interact with the ligand are shown as red spheres. (D) The overlap view of the ligand-binding site of StCysE. The Cys (cysteine) complex is shown in green and the Ser (serine) complex is shown in pink.

A

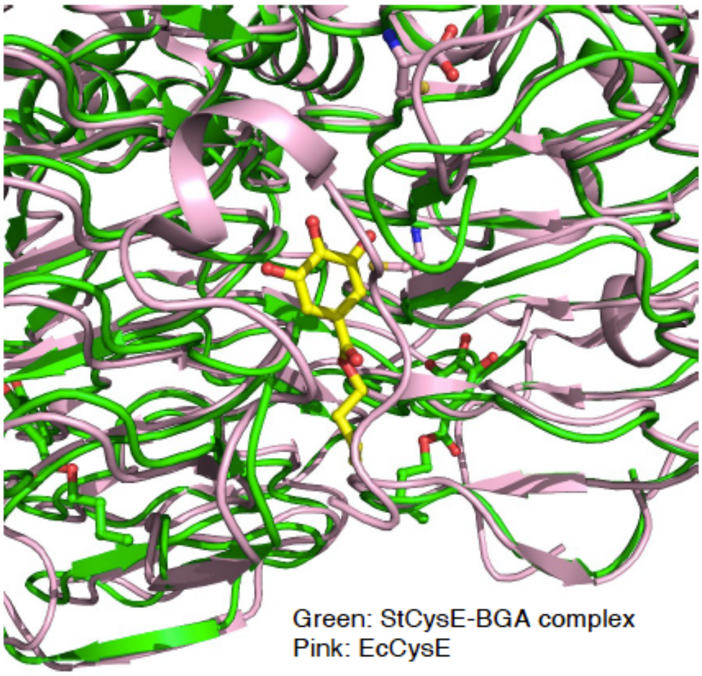

B

```
S_typhimurium      MHHHHHHDP---------PCEELEIVWKNIKAEARALADCEPMLASFYHATLLKHENLGS
E_Coli             -HHHHHHSSGLVPRGSHMSCEELEIVWNNIKAEARTLADCEPMLASFYHATLLKHENLGS

S_typhimurium      ALSYMLANKLASPIMPAIAIREVVEEAYAADPEMIASAACDIQAVRTRDPAVDKYSTPLL
E_Coli             ALSYMLANKLSSPIMPAIAIREVVEEAYAADPEMIASAACDIQAVRTRDPAVDKYSTPLL

S_typhimurium      YLKGFHALQAYRIGHWLWNKGRRALAIFLQNQVSVSFQVDIHPAAKIGRGIMLDHATGIV
E_Coli             YLKGFHALQAYRIGHWLWNQGRRALAIFLQNQVSVTFQVDIHPAAKIGRGIMLDHATGIV

S_typhimurium      VGETAVIEDDVSILQSVTLGGTGKTSGDRHPKIREGVMIGAGAKILGNIEVGRGAKIGAG
E_Coli             VGETAVIENDVSILQSVTLGGTGKSGGDRHPKIREGVMIGAGAKILGNIEVGRGAKIGAG

S_typhimurium      SVVLQPVPPHTTAAGVPARIVGKPGSDKPSMDMDQHFNGIHHTFEYGDGI
E_Coli             SVVLQPVPPHTTAAGVPARIVGKPDSDKPSMDMDQHFNGINHTFEYGDGI
```

Supplementary Figure 13. Structural comparison between *E. coli* CysE (EcCysE) and *S.* Typhimurium CysE (StCysE). (A) The overall structures. (B) Amino acid sequences. All amino acid residues that make up the binding site of BGA are conserved between StCysE and EcCysE. Amino acid residues marked with triangles represent those interacting with BGA (chain A) (Green, chain A; red, chain C).

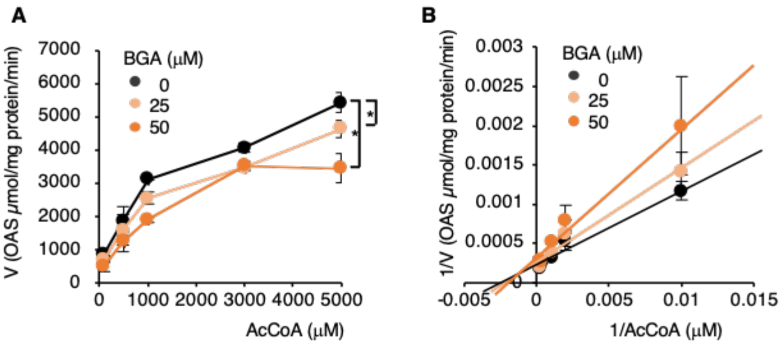

Supplementary Figure 14. Kinetic analyses of CysE reactions. (A) Enzyme kinetics of CysE with acetyl-CoA as a substrate in the presence of BGA. Reactions were carried out by using 10 µg/mL CysE, 5 mM serine, and acetyl-CoA (0, 0.1, 0.5, 1, 3 and 5 mM) in 100 mM NaPB (pH 7.6) at 37 °C for 1 min in the presence of BGA at the indicated concentrations. (B) The Lineweaver-Burk plot of A. Data are means ± SD (*n* =3). *, $p < 0.01$.

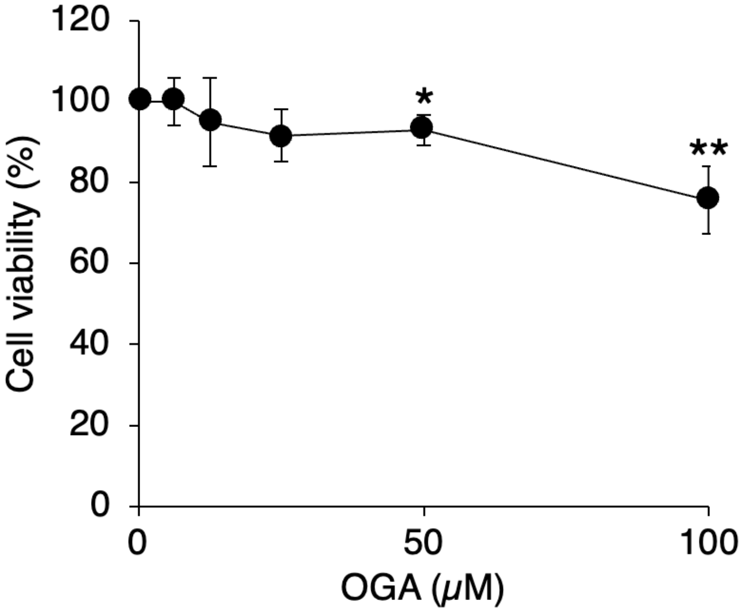

Supplementary Figure 15. Effect of OGA on cell viability of HEK293T cells. HEK293T cells were treated with indicated concentrations of OGA for 8 h. Cell viability was determined by means of MTT assay. Data are means ± SD (*n* =3). *, $p < 0.05$; **, $p < 0.01$.

Supplementary Table 1. MIC values of antibiotics that are effective against C.I *E.coli*-2.

PIPC	TAZ/ PIPC	CFPM	CZOP	GM	MINO	DRPM	AMK	LVFX	AZT	IPM
>64<br>R	>4/64<br>R	>16<br>R	>16	4<br>S	>8<br>R	2<br>I	>32<br>R	>4<br>R	2<br>S	2<br>I

IPM/ DPA	MEPM	MEPM/ DPA	CAZ	CAZ/ DPA	S/C	CL	FOM	TOB	CPFX	ST
1>	4<br>R	1>	>32<br>R	2	>32/32	>1	32	>8<br>R	>2<br>R	>38/2<br>R

CLSI M100-S22 category: S, sensitive; I, intermediate; R, resistant
Abbreviations: PIPC, piperacillin; TAZ, tazobactam; CFPM, cefepime; CZOP, cefozopran; GM, gentamycin; MINO, minocycline; DRPM, doripenem; AMK, amikacin; LVFX, levofloxacin; AZT, aztreonam; IPM, imipenem; DPA, dipicolinic acid; MEPM, meropenem; CAZ, ceftazidime; S/C, sulbactam/cefoperazone; CL, colistin; FOM, fosfomycin; TOB, tobramycin; CPFX, ciprofloxacin; and ST, sulfamethoxazole/trimethoprim. Antibiotic concentrations, μg/mL.

Supplementary Table 2. MIC values of antibiotics that are effective against C.I *K. pneumoniae*.

PIPC	TAZ/ PIPC	CFPM	CZOP	GM	MINO	DRPM	AMK	LVFX	AZT	IPM
>64 R	>4/64 R	>16 R	>16	4 S	8 I	>8 R	4 S	0.5 S	2 S	>16 R

IPM/ DPA	MEPM	MEPM/ DPA	CAZ	CAZ/ DPA	S/C	CL	FOM	TOB	CPFX	ST
1>	>16 R	1>	>32 R	2	>32/32	2>	>128	>8 R	0.5 S	>38/2 R

CLSI M100-S22 category: S, sensitive; I, intermediate; R, resistant
Abbreviations: PIPC, piperacillin; TAZ, tazobactam; CFPM, cefepime; CZOP, cefozopran; GM, gentamycin; MINO, minocycline; DRPM, doripenem; AMK, amikacin; LVFX, levofloxacin; AZT, aztreonam; IPM, imipenem; DPA, dipicolinic acid; MEPM, meropenem; CAZ, ceftazidime; S/C, sulbactam/cefoperazone; CL, colistin; FOM, fosfomycin; TOB, tobramycin; CPFX, ciprofloxacin; and ST, sulfamethoxazole/trimethoprim. Antibiotic concentrations, μg/mL.

Supplementary Table 3. MRM parameters for APDS-amino acid adducts

Analyte	Precursor ion (*m/z*)	Product ion (*m/z*)	Fragmentor voltage (V)	Collision energy (eV)	Polarity
APDS-lysine	387.2	147.2	90	17	+
APDS-tryptophan	325.1	121.2	90	29	+
APDS-tyrosine	302.1	121.2	90	25	+
APDS-arginine	295.2	175.3	90	13	+
APDS-phenylalanine	286.1	121.2	90	17	+
APDS-histidine	276.1	156.2	50	9	+
APDS-methionine	270.1	121.2	90	17	+
APDS-glutamic acid	268.1	121.2	90	21	+
APDS-glutamine	267.1	121.2	90	29	+
APDS-aspartic acid	254.1	121.2	90	21	+
APDS-asparagine	253.1	121.2	90	17	+
APDS-leucine	252.1	121.2	90	17	+
APDS-isoleucine	252.1	86.4	90	17	+
APDS-threonine	240.1	121.2	90	17	+
APDS-valine	238.1	121.2	90	17	+
APDS-proline	236.1	121.2	90	25	+
APDS-serine	226.1	121.2	90	17	+
APDS-alanine	210.1	121.2	90	17	+
APDS-glycine	196.1	121.2	90	13	+

Supplementary Table 4. Data collection and refinement

Data set	Cys-bound	Ser-bound	CoA-bound	Butyl gallate-bound
Data collection				
Diffraction source	SPring-8 BL44XU	SPring-8 BL44XU	SPring-8 BL44XU	SPring-8 BL44XU
Wavelength (Å)	0.90000	0.90000	0.90000	0.90000
Camera distance (mm)	250.0	250.0	360.0	210.0
Rotation range per image (°)	0.1	0.1	0.1	0.1
Total rotation range (°)	270	270	180	180
Exposure time per image (s)	0.1	0.1	0.1	0.1
Detector	EIGER X 16M	EIGER X 16M	EIGER X 16M	EIGER X 16M
Space group	$P3_221$	$P3_221$	$I2_13$	$C2$
a, b, c (Å)	121.9, 121.9, 127.7	122.2, 122.2, 128.2	161.7, 161.7, 161.7	177.4, 102.6, 158.7
α, β, γ (°)	90.0, 90.0, 120.0	90.0, 90.0, 120.0	90.0, 90.0, 90.0	90.0, 111.9, 90.0
Resolution range (Å)	50-1.9 (2.01 -1.9)	50-2.3 (2.44-2.3)	50-2.5 (2.65-2.5)	50.0-1.80 (1.90-1.80)
No. of molecules/AU	3	3	2	12
Total no. of reflections	1,311,661	737,080	747,786	854,353
No. of unique reflections	86,865	47,207	24,563	233,218
Completeness (%)	99.8 (98.8)	99.8 (99.0)	99.9 (99.8)	99.3 (97.8)
Multiplicity	15.1 (14.4)	15.6 (15.4)	30.4 (29.5)	3.7 (3.6)
$I/\sigma(I)$	24.7 (5.5)	14.4 (2.7)	26.0 (2.8)	7.2 (1.2)
$CC_{1/2}$	0.999 (0.867)	0.999 (0.878)	1.000 (0.861)	0.995 (0.813)
$R_{r.i.m.}$	0.070 (0.565)	0.103 (0.817)	0.102 (1.342)	0.102 (1.013)
Refinement				
Resolution (Å)	20-1.9	20-2.3	20-2.5	20-1.8
R_{work}/R_{free}	0.171 / 0.189	0.172 / 0.210	0.190 / 0.232	0.205 / 0.241
No. of non-hydrogen atoms				
Protein	5,796	5,803	3,631	22,149
Ligand	21 (Cys)	21 (Ser)	14 (Cys), 96 (CoA)	84 (Cys), 192 (BG)
Solvent	204	69, 5 (Pi)	9	734, 20 (Pi)
Average B-factors (Å^2)				
Protein	38.3	57.1	77.4	39.0
Ligand	29.2 (Cys)	42.7 (Ser)	128.3 (Cys), 95.6 (CoA)	95.6 (Cys), 50.5 (BG)
Solvent	35.7	46.3, 72.5 (Pi)	58.2	34.3, 106.7 (Pi)
RMSD				
Bond length (Å)	0.010	0.010	0.012	0.010
Bond angle (°)	1.423	1.445	1.714	1.491
Ramachandran plot (%)				
Favored regions	91.4	90.3	86.3	90.3
Allowed regions	8.1	9.1	13.5	9.1
Outers	0.5	0.6	0.2	0.6
PDB code	7E3Y	-	-	-
